# Regenerative Hair Pigmentation via Skin Organoids: Adaptive Patterning Mediated by Collagen VI and Semaphorin 3C

**DOI:** 10.1002/advs.202502436

**Published:** 2025-07-03

**Authors:** Tingting Li, Xinzhu Li, Xiao Xiang, Jundong Huang, Xinyu Shen, Mengyue Wang, Jingwei Jiang, Shiwen Shao, Zeming Li, Tian Xie, Deming Liu, Yiping Zhao, Rui Ma, Wenyu Wu, Wei Shi, Cheng‐Ming Chuong, Ji Li, Mingxing Lei

**Affiliations:** ^1^ Key Laboratory of Biorheological Science and Technology of Ministry of Education & 111 Project Laboratory of Biomechanics and Tissue Repair College of Bioengineering Chongqing University Chongqing 400044 China; ^2^ Department of Dermatology Xiangya Hospital Central South University Changsha 410083 China; ^3^ Department of Dermatology The First Hospital of China Medical University NHC Key Laboratory of Immunodermatology (China Medical University) Shenyang Liaoning 110001 China; ^4^ Division of Dermatology Huashan Hospital Fudan University Shanghai Institute of Dermatology Shanghai 200040 China; ^5^ Department of Pathology Keck School of Medicine University of Southern California Los Angeles CA 90033 USA

**Keywords:** adaptive patterning, fibroblast, hair follicle stem cells, melanocyte, skin organoids

## Abstract

The color patterns of mammalian fur are determined by melanocytes' ability to respond to and adapt to microenvironmental cues. However, these patterns can be lost following injury or under pathological conditions, and the underlying biological mechanisms remain poorly understood. In this study, reconstituted hair‐bearing skin is generated using skin organoids derived from dissociated epidermal cells, dermal cells, and melanocyte progenitors. The reconstituted skin exhibited pigmented hair patterns. By investigating the molecular cues involved in re‐establishing pigment patterns, it is demonstrated that this process is regulated through a two‐step mechanism. First, during skin organoid culture, signaling from dermal fibroblasts to melanocytes via the COL6A3‐CD44 pathway promotes the early maintenance of organotypic melanocytes. Subsequently, during hair follicle morphogenesis after skin organoid transplantation, signaling from the bulge to melanocytes via the SEMA3C‐NRP1 pathway regulates microtubule stability. This regulation guides melanocytes to migrate to their bulge stem cell niche, thereby enhancing hair pigmentation by promoting the adaptive patterning of melanocytes within the hair follicle. The study reveals two novel signaling mechanisms that shape melanocyte adaptive patterning and highlight the hair follicle as a regulatory hub for melanocyte physiological behaviors. These findings may inspire new clinical strategies for preventing hair greying.

## Graphical abstract

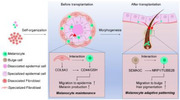

## 1 Introduction

Animals have evolved a rich variety of pelage colors that confer unique adaptive advantages, such as evading predators, engaging in courtship displays, and regulating body temperature.^[^ ^1^ ^]^ The biological principles behind the formation of these pigmentation patterns have long been a fascinating question for scientists. In developing skin, melanocytes originate from neural crest cells (NCCs), a multipotent, migratory cell population that emerges during early vertebrate embryogenesis.^[^ ^2^ ^]^ The specification of melanocyte precursors, known as melanoblasts, is orchestrated by a network of transcription factors, including SRY‐Box transcription factor 10 (SOX10),^[^ ^3^ ^]^ paired box gene 3 (PAX3),^[^ ^4^ ^]^ and microphthalmia‐associated transcription factor (MITF).^[^ ^5^ ^]^ Once specified, melanoblasts begin their migration from the neural crest to their destined anatomical site, such as the skin.^[^ ^6^ ^]^ Upon reaching the dermis, melanoblasts continue to proliferate and differentiate while traverse into the epidermis where they enter the hair follicle after the formation of dermal condensation.^[^ ^7^ ^]^ Within the hair follicle, melanocytes primarily colonize the bulge region, which serves as the reservoir containing melanocyte stem cells, and the hair matrix (HM) that surrounds the dermal papilla, where differentiated melanocytes transport melanosomes to keratinocytes for hair pigmentation.^[^ ^8^, ^9^ ^]^

Intriguingly, melanocytes also show remarkable adaptability to external stimuli.^[^ ^10^ ^]^ For instance, the epidermis‐residing melanocytes serve a crucial protective role against the harmful effects of ultraviolet (UV) radiation. UV radiation can cause irreversible damage to cellular DNA, which prompts the activation of the p53 tumor‐suppressor protein leading to elevated melanin production.^[^ ^10^ ^]^ Thus, the biological principles that govern melanocytic pigmentation patterns are complex, and understanding the precise mechanisms behind their self‐organization and adaptive behaviors may inspire us to devise novel therapeutic approaches to treat human diseases such as premature hair graying and vitiligo.

As a 3D culture system, skin organoids can simulate the complex structure and function of the skin, providing an important platform for studying the physiological and pathological mechanisms.^[^ ^11^, ^12^ ^]^ In recent years, skin organoids have offered unique advantages in exploring cell‐cell interactions and tissue self‐organization because of their ability to highly mimic the cellular composition and spatial structure of the skin in vivo. In addition, skin organoids can self‐organize and reproduce structures in the body, thus it is an excellent model for studying the dynamic tissue positioning melanocytes.^[^ ^13^, ^14^, ^15^, ^16^ ^]^ Indeed, our previous studies have shown that transplanting skin‐derived organoids from mice with different hair color varieties into nude mice can regenerate hair of the same color as the donor. This further demonstrates the potential of skin organoids to simulate key steps in melanocyte development.^[^ ^13^ ^]^ However, how melanocytes organize from a completely random state during the skin organoid formation to ultimately position themselves at the set anatomical locations and generate the adaptive patterning to pigment the regenerated hairs after transplantation still requires extensive research.

In this study, we compared the behavior of melanocytes during hair follicle formation between embryonic development and skin organoids. We then used skin organoids as a model to elucidate the mechanisms underlying pigment patterning. We found that the spatial and temporal localization of melanocytes in mouse skin organoids closely mimics that observed under physiological conditions. Specifically, COL6A3 (Collagen type VI alpha 3 chain) secreted by fibroblasts and SEMA3C (Semaphorin 3C) released from the bulge region orchestrate melanocyte behavior through a two‐step mechanism. First, COL6A3 promotes melanocyte maintenance during skin organoid culture. Subsequently, SEMA3C enhances adaptive melanocyte patterning during skin organoid morphogenesis and hair regeneration. This sequential regulation provides a foundation for understanding the mechanisms of hair pigment regulation and may inform therapeutic strategies for treating pigmentation disorders and advancing regenerative medicine.

## 2 Results

### 2.1 The Cellular Processes Underlying Melanocyte Adaptive Patterning in Skin Organoid‐Based Hair Regeneration Closely Parallel Those Observed During Embryonic Skin Development

**Figure 1:**
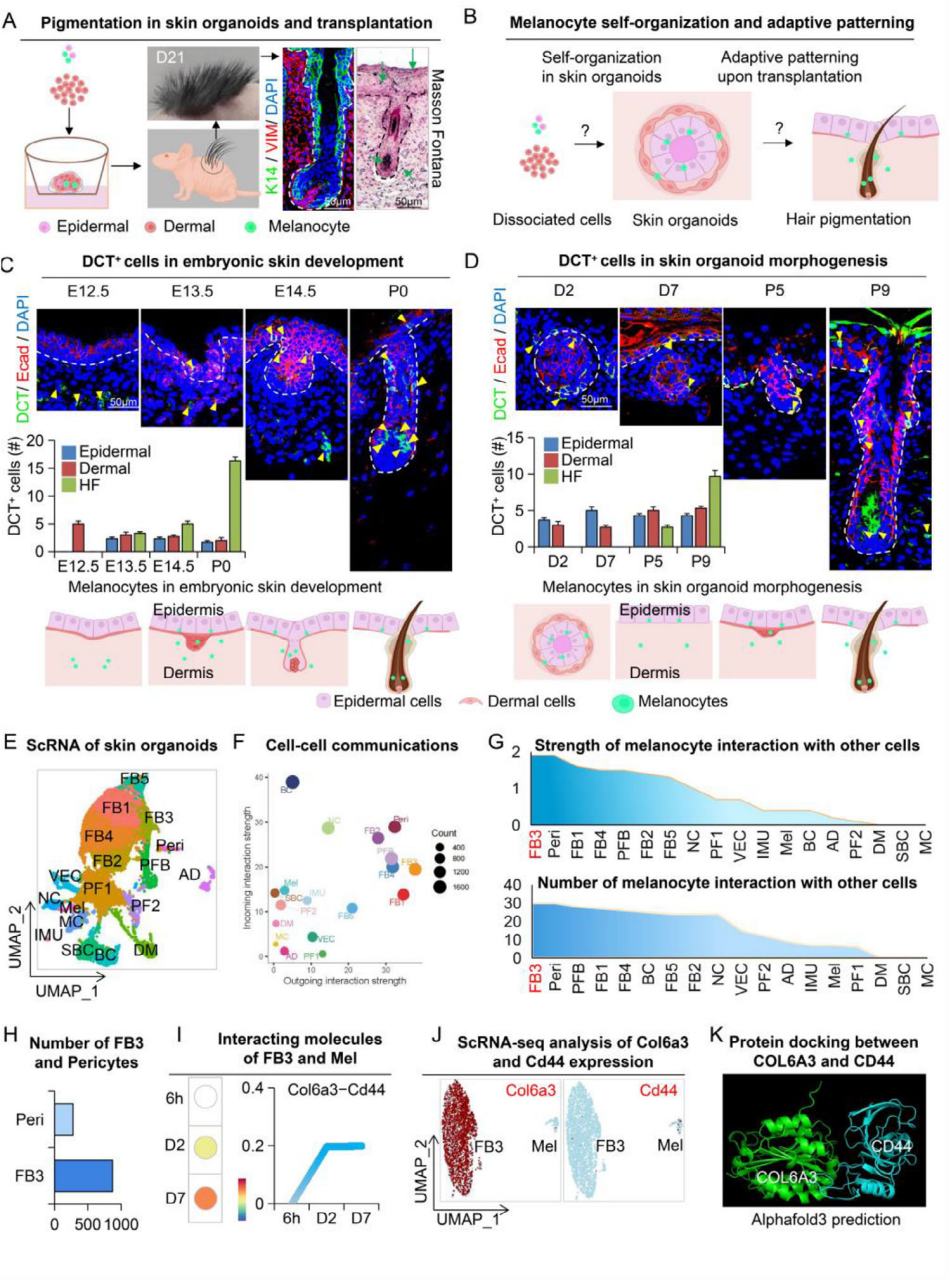
Spatiotemporal localization of the melanocyte lineage during development in vivo and in skin organoids in vitro A) Schematic representation of in vitro culture and in vivo transplantation of C57BL/6 mouse skin organoids. Scale b ar = 50 µm. B) Visualization of melanocyte self‐organization in skin organoids and their adaptive behavior after transplantation. C) Patterning of melanocyte progenitors toward the hair follicles in developing skin. Immunofluorescence staining and quantification for DCT, E‐cad (E‐cadherin), and DAPI. Quantification and a graphic summary are shown below. N ≥ 3, scale bar = 50 µm. D) Patterning of melanocyte progenitors toward the hair follicles in reconstituted skin derived from skin organoids. Similar immunofluorescence. Quantification and a graphic summary are shown below for comparison. N ≥ 3, scale bar = 50 µm. E) UMAP plot of skin organoids at 6 h, Day 2, and Day 7, showing unbiased clustering. F) Signal pathway analysis of cell‐cell communication networks shows stronger signal output from FB3. G) Statistical analysis of interaction numbers and signal strength among all cell types. H) Quantification of FB3 cells and pericytes. I) Quantitative analysis of Col6a3‐Cd44 interaction intensity between FB3 cells and melanocytes over different time points. J) UMAP plots showing Col6a3 and CD44 expression in FB3 and melanocyte clusters respectively by unbiased clustering. K) Protein docking analysis using Alphafold3 shows the interaction of COL6A3 and CD44.

Skin organoids provide a versatile and convenient in vitro platform for studying the development of skin and its appendages. We have previously developed a method for constructing skin organoids using mixtures of epidermal and dermal cells extracted from murine skin.^[^ ^12^ ^]^ The mixture of cells used in our experiments inevitably included in situ melanocytes. Surprisingly, skin organoids derived from neonatal C57BL/6 (black) mice produced pigmented hair 21 days after transplantation into hairless nude mice (**Figure** **1**A). More remarkably, immunostaining and Masson‐Fontana staining of the transplanted skin samples revealed that pigmentation of the skin and hair follicles began as early as 9 days post‐transplantation. In contrast, skin organoids generated in parallel from CD1 mice (white) did not show any signs of pigmentation, even though they developed into hair‐producing skin on the recipient nude mice (Figure S1A, Supporting Information). This observation indicates that melanocytes are not only functionally active but also highly responsive to local cues for spatial positioning (Figure 1B). However, the mechanisms underlying these adaptive patterning processes remain to be elucidated.

To unravel this biological puzzle, we first employed melanocyte‐specific markers Dopachrome tautomerase (DCT) and Tyrosinase (TYR) to track melanocyte localization in the skin of C57BL/6 mice at various embryonic developmental stages (Figure 1C; Figure S1B, Supporting Information). These time points were specifically chosen as they represent the critical morphogenetic stages of hair follicles (reference). We found that melanocytes were initially located in the dermis at embryonic day 12.5 (E12.5) but began migrating to the epidermis by E13.5. By E14.5, epidermal melanocytes clustered around the dermal condensation as hair follicles developed. At postnatal day 0 (P0), melanocytes were scarcely present in the dermis and epidermis; instead, they were predominantly detected within fully developed hair follicles, particularly in the bulge and hair matrix (Figure 1C; Figure S1B, Supporting Information).

Interestingly, the spatial organization of melanocytes in skin organoids closely mirrored that observed during embryonic development. During in vitro organoid development, melanocytes were initially detected in both the epidermal (inner) and dermal (outer) layers on day 2 (D2) but began to preferentially localize to the epidermal layers by day 7 (D7), resembling the pattern seen at E13.5 (Figure 1D; Figure S1E, Supporting Information). After transplantation into nude mice, melanocytes appeared in developing hair follicles on postnatal day 5 (P5), similar to the pattern at E14.5. By P9, when hair follicles were fully formed, melanocytes were more widely distributed, including in the dermis and epidermis, but were still most abundant in the bulge and hair matrix, closely reflecting the distribution seen at P0 (Figure 1D; Figure S1E, Supporting Information). These results demonstrate a striking similarity in melanocyte behavior between skin organoids and embryonic development.

### 2.2 Identifying Crosstalk Between Melanocytes and Fibroblasts in Skin Organoids

To decipher the cues of melanocyte adaptive behavior in organoids, we used single‐cell RNA sequencing (scRNA‐seq) data of skin organoids at 6h, D2, and D7, which represented 3 critical stages of morphogenesis, namely, dissociation, polarization, and planarization, respectively.^[^ ^12^ ^]^ We identified 18 unique cell populations using a set of specific marker genes including dermal cells (FB1‐5), epidermal cells (SBC and BC), and melanocytes (Mel) (Figure 1E; Figure S1F,G, Supporting Information). As studies have shown that Col2a1 ablation in adult mouse epidermal stem cells results in epidermal hyperpigmentation due to the dramatic accumulation of melanocytes within the interfollicular epidermis,^[^ ^20^ ^]^ we hypothesized that ECM (extracellular matrix) components may regulate melanocyte self‐organization in mouse skin organoids. Therefore, we analyzed the scRNA‐seq data using CellChat for the potential involvement of ECM‐receptor interaction.^[^ ^21^ ^]^ Notably, our analysis revealed that Mel interacted most strongly with FB3 (fibroblasts) and Peri (pericytes) (Figure 1F) between which FB3 had a stronger predicted interaction (Figure 1G; Figure S1H, Supporting Information) and was more abundant (Figure 1H). Further bioinformatic analysis showed COLLAGEN was the strongest ligand from FB3 and specifically Col6a3 was predicted to interact with receptor Cd44 on Mel (Figure S1I.J, Supporting Information). Interestingly, the interaction strength of the Col6a3‐Cd44 ligand/receptor pair increased as skin organoids developed similar to the previously observed temporal interaction between melanocytes and epidermal cells (Figure 1I). Moreover, Col6a3 and Cd44 expression was detected in FB3 and Mel, respectively (Figure 1J), and their protein products were predicted by Afold3 to physically interact (Figure 1K), thus warranting further investigation.

### 2.3 COL6A3‐CD44 Signaling Maintains Melanocytes In Vivo

**Figure 2:**
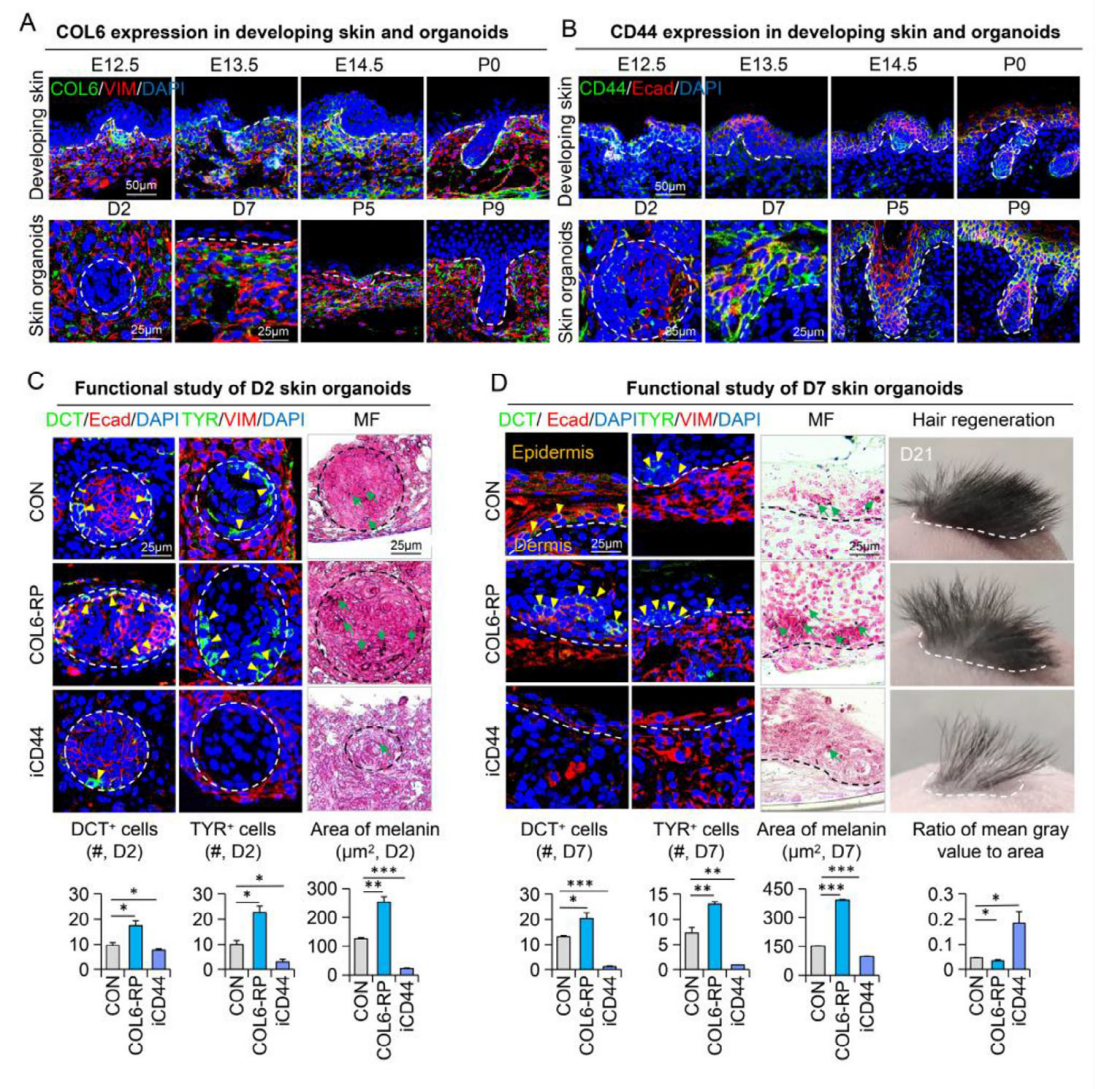
Functional characterization of the COL6A3‐CD44 axis in regulating melanocyte organization and pigmentation A) COL6A3 expression in embryonic skin and mouse skin organoids. Developing skin: scale bars = 50 µm. Skin organoids: scale bar = 25 µm. N ≥ 3. B) CD44 expression in embryonic skin and mouse skin organoids. Developing skin: scale bars = 50 µm. Skin organoids: scale bar = 25 µm. N ≥ 3. C) Masson‐Fontana staining and immunofluorescence staining of DCT, E‐cad, DAPI, TYR, and VIMENTIN in Day 2 skin organoids from the control group, COL6A3‐activated group, and CD44‐inhibited group. Quantifications of DCT+, and TYR+ cell numbers as well as melanin positive area are shown below. N ≥ 3; ^*^ *p* < 0.05, ^**^ *p* < 0.01, ^***^ *p* < 0.001; scale bar = 25 µm. D) Masson‐Fontana staining and immunofluorescence staining of DCT, E‐cad, DAPI, TYR, and VIMENTIN in Day 7 skin organoids and Day 21 after transplantation into nude mice. Quantifications of DCT+ and TYR+ cell numbers, and the ratio of mean gray value to area on Day 21 post‐transplantation melanin positive area are shown below. N ≥ 3; ^*^ *p* < 0.05, ^**^ *p* < 0.01, ^***^ *p* < 0.001; scale bar = 25 µm.

We then assessed COL6 and CD44 expression during both embryonic development and skin organoid morphogenesis by immunostaining. Our results revealed that, in both models, COL6 and CD44 were distinctively detected in the dermal and epidermal compartments, respectively, albeit CD44 is also expressed in other epidermal cells (**Figure** **2**A,B). Therefore, we hypothesized that COL6 in the dermal fibroblasts might interact with the CD44 receptors on melanocytes, thus influencing their spatial patterning and function. To test this, we modulated the COL6A3‐CD44 interaction in skin organoids using human COL6A3 recombinant protein (COL6A3‐RP) or Angstrom6, a potent CD44 inhibitor (iCD44), and observed the behaviors of melanocytes by immunostaining.^[^ ^22^ ^]^ On both D2 and D7, the addition of COL6A3‐RP significantly increased the number of melanocytes and promoted localized melanin deposition compared with the control group (Figure 2C,D). On the contrary, inhibition of CD44 resulted in the opposite effects (Figure 2C,D). Moreover, the same observations were also made at other chosen time points (Figure S2A,B, Supporting Information). More importantly, when the treated skin organoids were transplanted in nude mice, COL6A3‐RP induced the growth of hyperpigmented hair while inhibition of CD44 led to both a loss of pigmentation and a reduction in hair growth. These results strongly suggest a regulatory role of COL6A3 on melanocyte migration and function.

**Figure 3:**
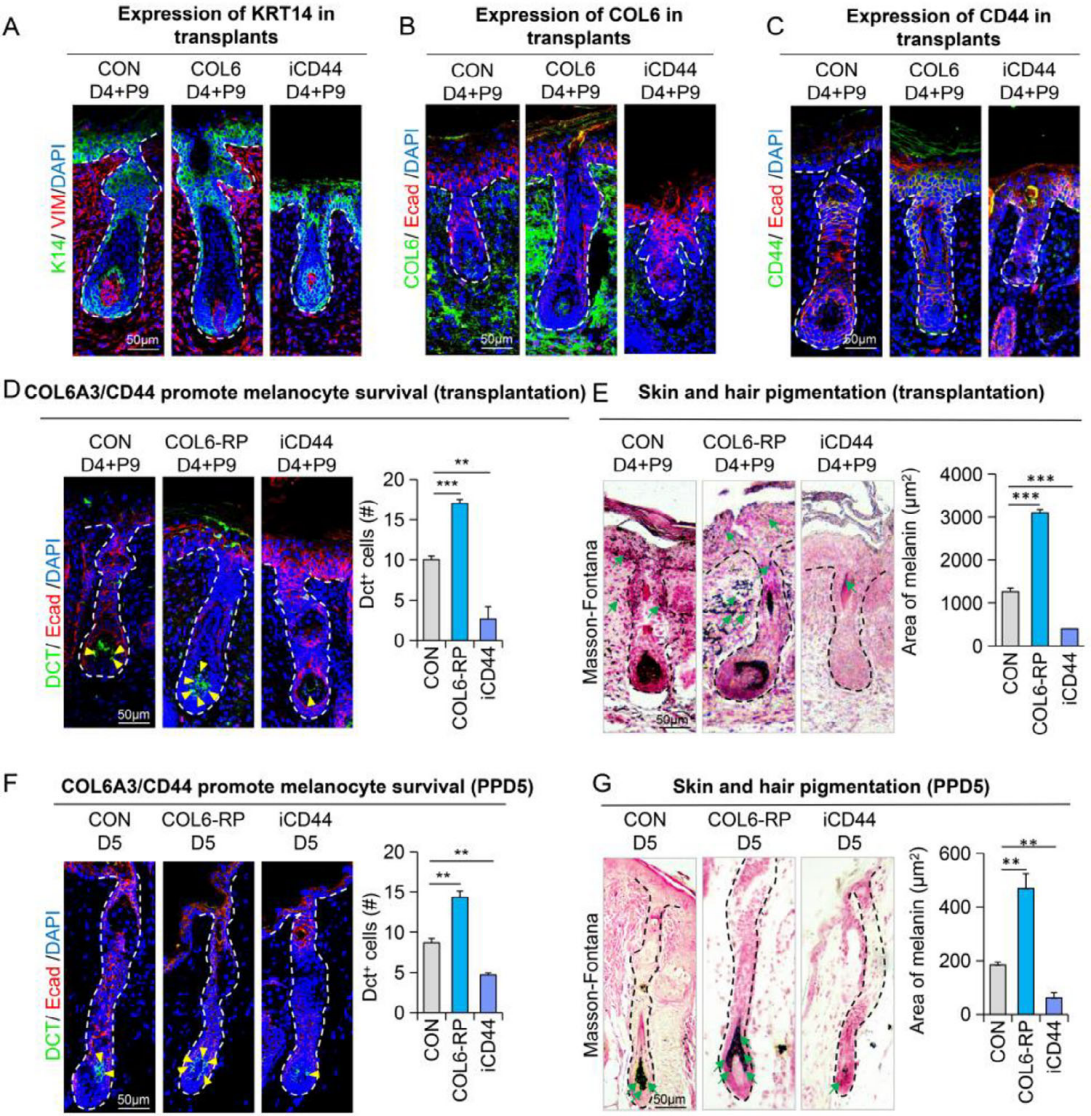
COL6A3‐CD44 signaling maintains melanocytic population and regulates hair pigmentation during skin organoids morphogenesis A) Immunofluorescence staining for K14 (KRT14), VIMENTIN, and DAPI in skin organoids from the control group, COL6A3‐activated group, and CD44‐inhibited group at 9 days post‐transplantation. Scale bar = 50 µm. B) Immunofluorescence staining for COL6, E‐cad, and DAPI in the same group as in (A) Scale bar = 50 µm. C) Immunofluorescence staining for CD44, E‐cad, and DAPI in the same groups as in (A) Scale bar = 50 µm. D) Immunofluorescence staining for DCT, E‐cad, and DAPI, with quantification of DCT+ cells in each group. N ≥ 3; ^**^ *p* < 0.01, ^***^ *p* < 0.001; scale bar = 50 µm. E) Masson‐Fontana staining and quantification of melanin area in each group. N ≥ 3; ^***^ *p* < 0.001; scale bar = 50 µm. F) Immunofluorescence staining for DCT, E‐cad, and DAPI, with quantification of DCT+ cells in each group at Day 5 in plucked mice. N ≥ 3; ^**^ *p* < 0.01; scale bar = 50 µm. G) Masson‐Fontana staining and quantification of melanin area in each group at Day 5 in plucked mice. N ≥ 3; ^**^ *p* < 0.01; scale bar = 50 µm.

Mature melanocytes are primarily located in the hair matrix region, where they transport melanin to keratinocytes to pigment hair shafts. To further investigate this process, we collected hair follicle samples from the transplanted skin (Figure S3A, Supporting Information). As anticipated, treatment with COL6A3‐RP (recombinant protein) promoted hair follicle regeneration, whereas inhibition of CD44 (via iCD44) suppressed it (**Figure** **3**A,C). Notably, COL6A3‐RP treatment significantly increased the number of melanocytes and the area of melanin deposits in the hair follicles, while CD44 inhibition had the opposite effect compared to controls (Figure 3D,E). We further examined the role of the COL6A3‐CD44 interaction during hair growth using a hair‐plucking mouse model (Figure S3C, Supporting Information). Consistent with our previous findings, administration of COL6A3‐RP led to increased accumulation of melanocytes and melanin deposits in the hair bulb, whereas CD44 inhibition significantly reduced these effects (Figure 3F,G; Figure S3D, Supporting Information). Collectively, these results demonstrate that COL6A3 derived from fibroblasts (FB3) regulates melanocyte trafficking, survival, and function by interacting with the CD44 receptor on melanocytes.

### 2.4 Glutathione Metabolism Functions Downstream of the Col6a3‐Cd44 Pathway

**Figure 4:**
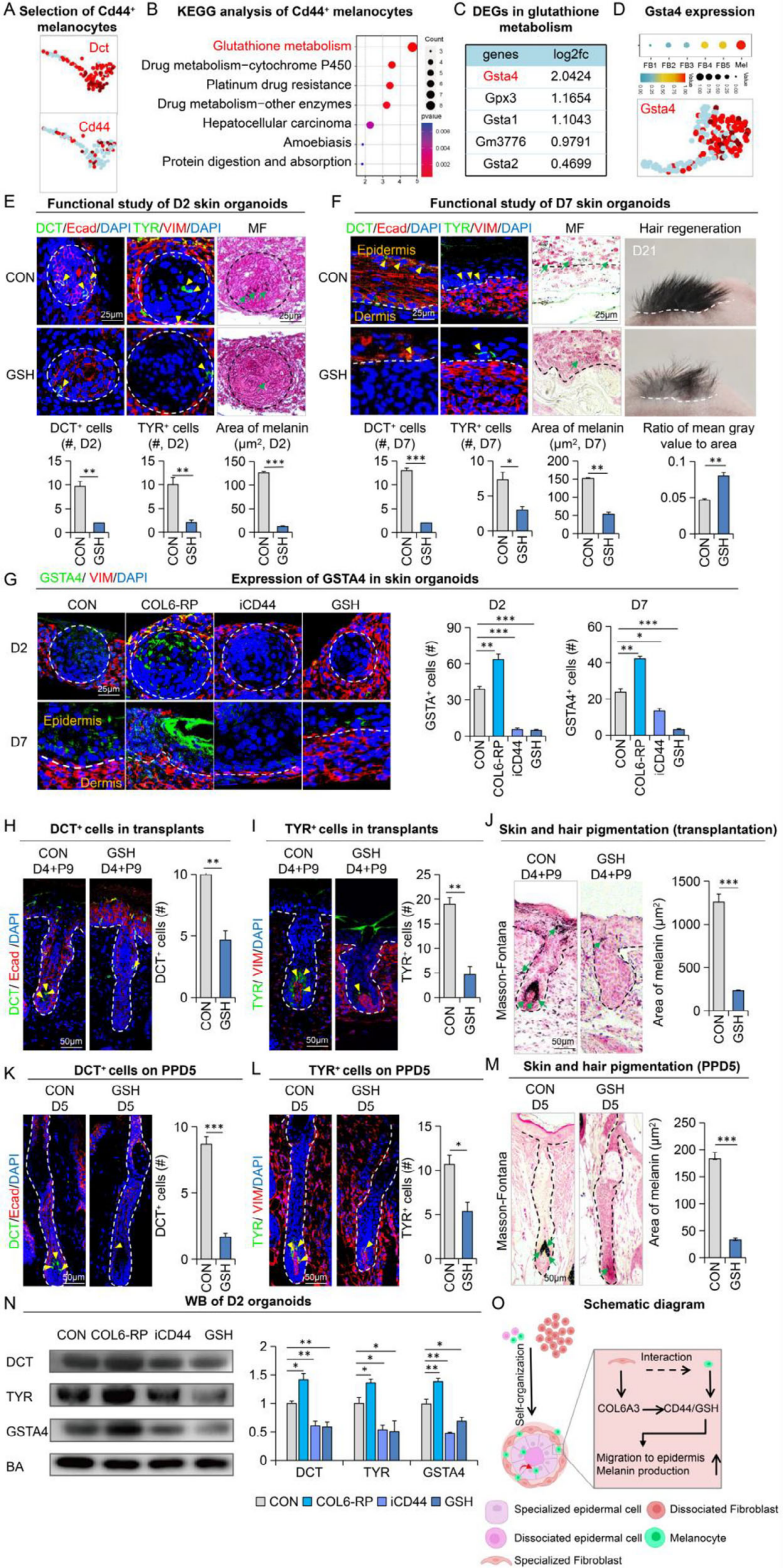
CD44‐mediated activation of the glutathione pathway enhances melanocytic niche and function A) UMAP plots showing DCT and CD44 expression in the melanocyte cluster by unbiased clustering. B) KEGG enrichment analysis of CD44+ cells within the melanocyte cluster. C) Differentially expressed genes in the glutathione metabolic pathway. D) scMetabolism analysis of melanocytes and fibroblasts, along with UMAP plots showing Gsta4 expression in the melanocyte cluster by unbiased clustering. E) Masson‐Fontana staining and immunofluorescence staining for DCT, E‐cad, DAPI, TYR, and VIMENTIN in Day 2 skin organoids from the control group and glutathione addition group. Quantification of melanin area, DCT+ cell count, and TYR+ cell count. N ≥ 3; ^**^ *p* < 0.01, ^***^ *p* < 0.001; scale bar = 25 µm. F) Masson‐Fontana staining and immunofluorescence staining for DCT, E‐cad, DAPI, TYR, and VIMENTIN in Day 7 skin organoids and those transplanted into nude mice for 21 days in the control and glutathione addition groups. Quantification of melanin area, DCT+ cell count, TYR+ cell count, and the ratio of mean gray value to area on Day 21 post‐transplantation. N ≥ 3; ^*^ *p* < 0.05, ^**^ *p* < 0.01, ^***^ *p* < 0.001; scale bar = 25 µm. G) Immunofluorescence staining for GSTA4, VIMENTIN, and DAPI, with quantification of GSTA4+ cells in the control group, COL6A3‐activated group, CD44‐inhibited group, and glutathione addition group on Days 2 and 7 of in vitro cultures. N ≥ 3; ^*^ *p* < 0.05, ^**^ *p* < 0.01, ^***^ *p* < 0.001; scale bar = 25 µm. H) Immunofluorescence staining for DCT, E‐cad, and DAPI, with quantification of DCT+ cells in skin organoids from the control and glutathione addition groups at 9 days post‐transplantation. N ≥ 3; ^**^ *p* < 0.01, scale bar = 50 µm. I) Immunofluorescence staining for TYR, VIMENTIN, and DAPI, with quantification of TYR+ cells in skin organoids from the control and glutathione addition groups at 9 days post‐transplantation. N ≥ 3; ^**^ *p* < 0.01, scale bar = 50 µm. J) Masson‐Fontana staining and quantification of the melanin area in skin organoids from the control and glutathione addition groups at 9 days post‐transplantation. N ≥ 3; ^***^ *p* < 0.001, scale bar = 50 µm. K) Immunofluorescence staining for DCT, E‐cad, and DAPI, with quantification of DCT+ cells in skin organoids from the control and glutathione addition groups on Day 5 in plucked mice. N ≥ 3; ^*^ *p* < 0.05, scale bar = 50 µm. L) Immunofluorescence staining for TYR, VIMENTIN, and DAPI, with quantification of TYR+ cells in skin organoids from the control and glutathione addition groups on Day 5 in plucked mice. N ≥ 3; ^*^ *p* < 0.05, scale bar = 50 µm. M) Masson‐Fontana staining and quantification of the melanin area in skin organoids from the control and glutathione addition groups on Day 5 in plucked mice. N ≥ 3; ^***^ *p* < 0.001, scale bar = 50 µm. Representative Western blot images and quantifications show up‐regulation of DCT, TYR, and GSTA by COL6‐RP and down‐regulation of these proteins after treatment with iCD44 and GSH in D2 mouse skin organoids. The results are shown as mean ± S.D., and were analyzed by One‐way ANOVA with Dunnett's post‐hoc test. ^*^ *p* < 0.05, ^**^ *p* < 0.01, and ^***^ *p* < 0.001. N=3. Schematic diagram of COL6A3‐CD44 axis.

To better understand how the COL6A3‐CD44 interaction influences the anatomical fate and function of melanocytes, we performed KEGG enrichment analysis on Cd44+ cells from the total melanocyte population (**Figure** **4**A). We found that the glutathione (GSH) metabolism pathway was the most significantly enriched pathway (Figure 4B), and among the related genes, Gsta4 exhibited the highest fold change (Figure 4C; Figure S4A,B, Supporting Information). Next, we extracted fibroblast (FB) and melanocyte (Mel) populations from the integrated data for ScMetabolism analysis, which revealed prominent GSH metabolism in melanocytes (Figure 4D). This led us to hypothesize that GSH metabolism might be critical for the survival and function of melanocytes.

To test this hypothesis, we treated skin organoids with GSH at different developmental stages and analyzed them by immunofluorescence. Our results showed that administration of exogenous GSH dramatically reduced the number of melanocytes and melanin secretion, confirming the predictions from our bioinformatic analyses (Figure 4E,F; Figure S4C,D, Supporting Information). More remarkably, GSH‐treated skin organoids produced non‐pigmented hair after transplantation into nude mice, further suggesting a loss of melanocytes or their function (Figure 4F).

We next assessed the change in overall GSTA4+ cells after GSH treatment. Consistent with our previous observations, the number of GSTA4+ cells in skin organoids increased after COL6A3‐RP treatment but was significantly reduced by CD44 inhibition (Figure 4G; Figure S4E, Supporting Information). Importantly, GSH treatment reduced the number of GSTA4+ cells to the same extent as CD44 inhibition, suggesting that excessive GSH may inhibit GSH metabolism by eliminating GSTA4+ cells, thereby compromising melanocyte survival.

To further demonstrate this, we compared the number of melanocytes and melanin deposition in hair follicles from skin organoid transplants and after plucking‐induced hair regrowth. As expected, we detected a decrease in GSTA4+ cell numbers after GSH treatment in both transplants and post‐plucking (Figure S4F,G, Supporting Information), and GSH also inhibited hair follicle growth (Figure S4G, Supporting Information). Notably, there was a sharp decline in melanocytes in the hair bulb following GSH treatment, resulting in significantly reduced melanin secretion in both transplants (Figure 4H–J) and after plucking (Figure 4K–M). These findings were also consistent with our Western blotting analyses (Figure 4N). Moreover, to exclude the involvement of alternative pathways, we assessed melanin deposition and melanin content in skin organoids cultured in vitro for 2 days using recombinant SCF protein (SCF‐RP)^[^ ^23^ ^]^ and COL1 inhibitor (iCOL1). SCF‐RP treatment increased melanocyte deposition, melanin content (Figure S4I, Supporting Information), and the number of GSTA4⁺ cells (Figure S4J, Supporting Information), while iCOL1 treatment showed no significant changes compared to controls. These findings were further supported by WB analysis (Figure S4K, Supporting Information). Collectively, these results suggest a close link between GSH metabolism and melanocyte survival (Figure 4O).

### 2.5 ScRNA‐Seq Analysis of Crosstalk Between Melanocytes and Bulge Cells

**Figure 5:**
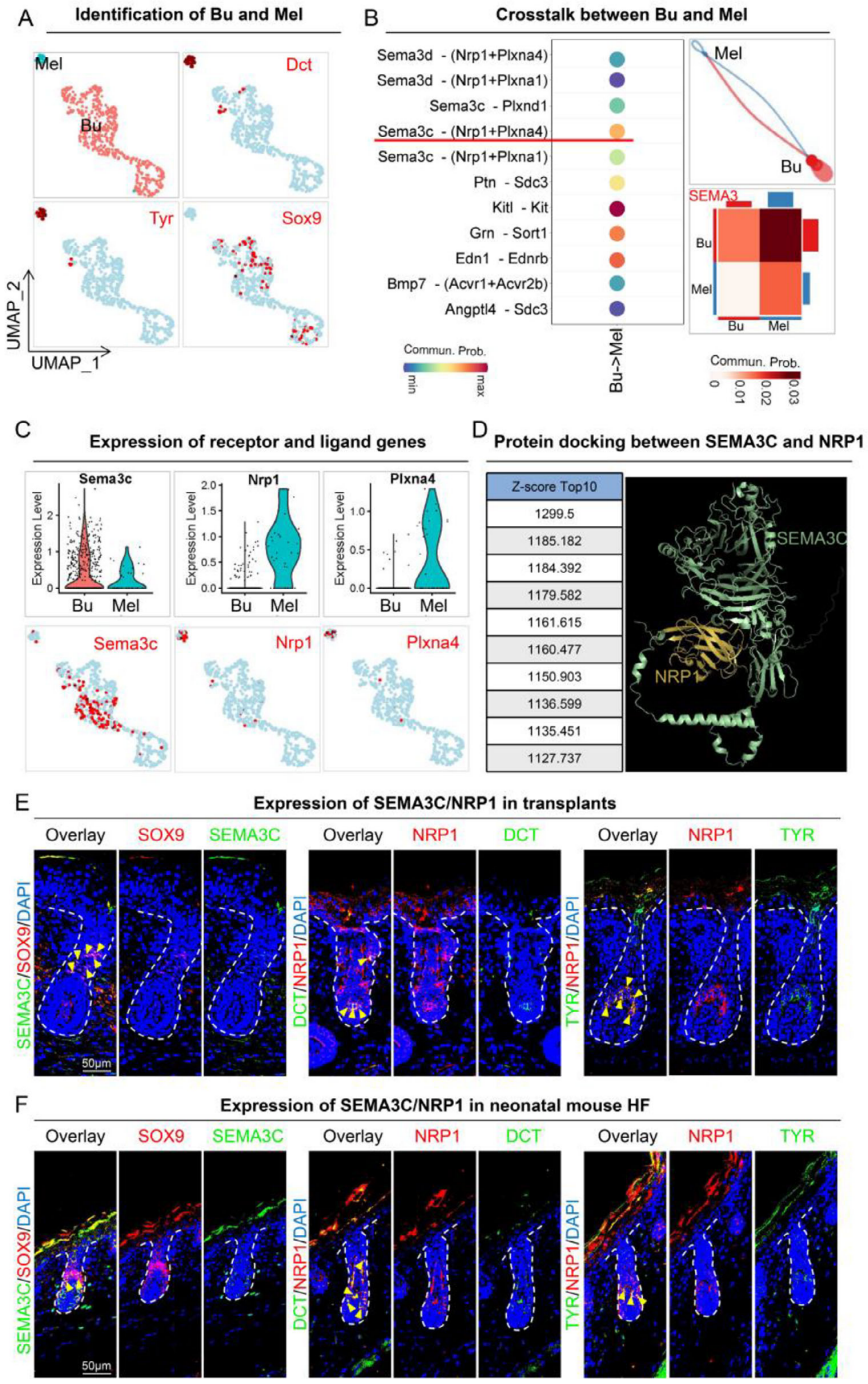
Crosstalk between melanocytes and bulge cells via the SEMA3C‐NRP1 axis enhances melanocyte recruitment and function A) UMAP plots showing Dct, Tyr, and Sox9 expression in melanocyte and bulge cell clusters by unbiased clustering. B) CellChat analysis of interactions between bulge cells and melanocytes. C) Violin plots and feature plots of Sema3c, Nrp1, and Plxna4 expression. D) Protein docking analysis of the interaction between SEMA3C and NRP1. E) Immunofluorescence staining for SEMA3C, SOX9, and DAPI, alongside DCT, NRP1, and TYR in skin organoids at 9 days post‐transplantation. Scale bar = 50 µm. F) Immunofluorescence staining for SEMA3C, SOX9, and DAPI, alongside DCT, NRP1, and TYR in neonatal mouse skin. Scale bar = 50 µm.

Melanocyte stem cells in the Bulge region (Bu) are capable of self‐renewal and differentiation to replenish the mature melanocyte pool and maintain tissue homeostasis, repair, and regeneration.^[^ ^24^ ^]^ To understand how melanocytes are first recruited to the Bulge region, we obtained neonatal mouse single‐cell RNA‐sequencing data and isolated the melanocytes (Dct+ and Tyr+) and Bulge‐resident stem cells (Sox9+) (**Figure** **5**A). Subsequently, we analyzed the potential molecular signals transmitted from the Bu to Mel using the CellChat with the Secreted Signaling add‐on.^[^ ^21^ ^]^ We found several prominent ligand‐receptor pairs that might facilitate the crosstalk between Bu and Mel, which were Kit‐Kitl, Edn1‐Ednrb, Grn‐Sort1, and Sema3c‐(Nrp1+Plxna4) (Figure 5B).

Further analyses showed that Sema3c‐(Nrp1+Plxna4) was specifically expressed and matched the expression pattern of genes known to be involved in melanocyte recruitment studies, such as Kit‐Kitl,^[^ ^25^ ^]^ Edn1‐Ednrb (Figure 5C; Figure S5A,B, Supporting Information).^[^ ^26^ ^]^ In addition, AlphaFold3‐aided protein docking^[^ ^27^ ^]^ of SEMA3C‐NRP1 and SEMA3C‐PLXNA4 showed a higher Z‐score for SEMA3C‐NRP1 (Figure 5D; Figure S5C, Supporting Information), suggesting that the interaction between this ligand‐receptor pair was more probable. Hence, we assessed the expression characteristics of SEMA3C and NRP1 in skin organoid transplants and hair follicles from neonatal mice. In both cases, we found a clear overlap of SEMA3C with SOX9 and NRP1 with DCT or TYR (Figure 5E,F; Figure S5D, Supporting Information), suggesting the expression of SEMA3C from Bulge cells and NRP1 from melanocytes, respectively. These data collectively indicate a possible functional relationship between Bulge cells and melanocytes through SEMA3C and NRP1 in a paracrine mechanism.

### 2.6 SEMA3C‐NRP1 Promotes the Recruitment of Melanocytes to the Bulge

**Figure 6:**
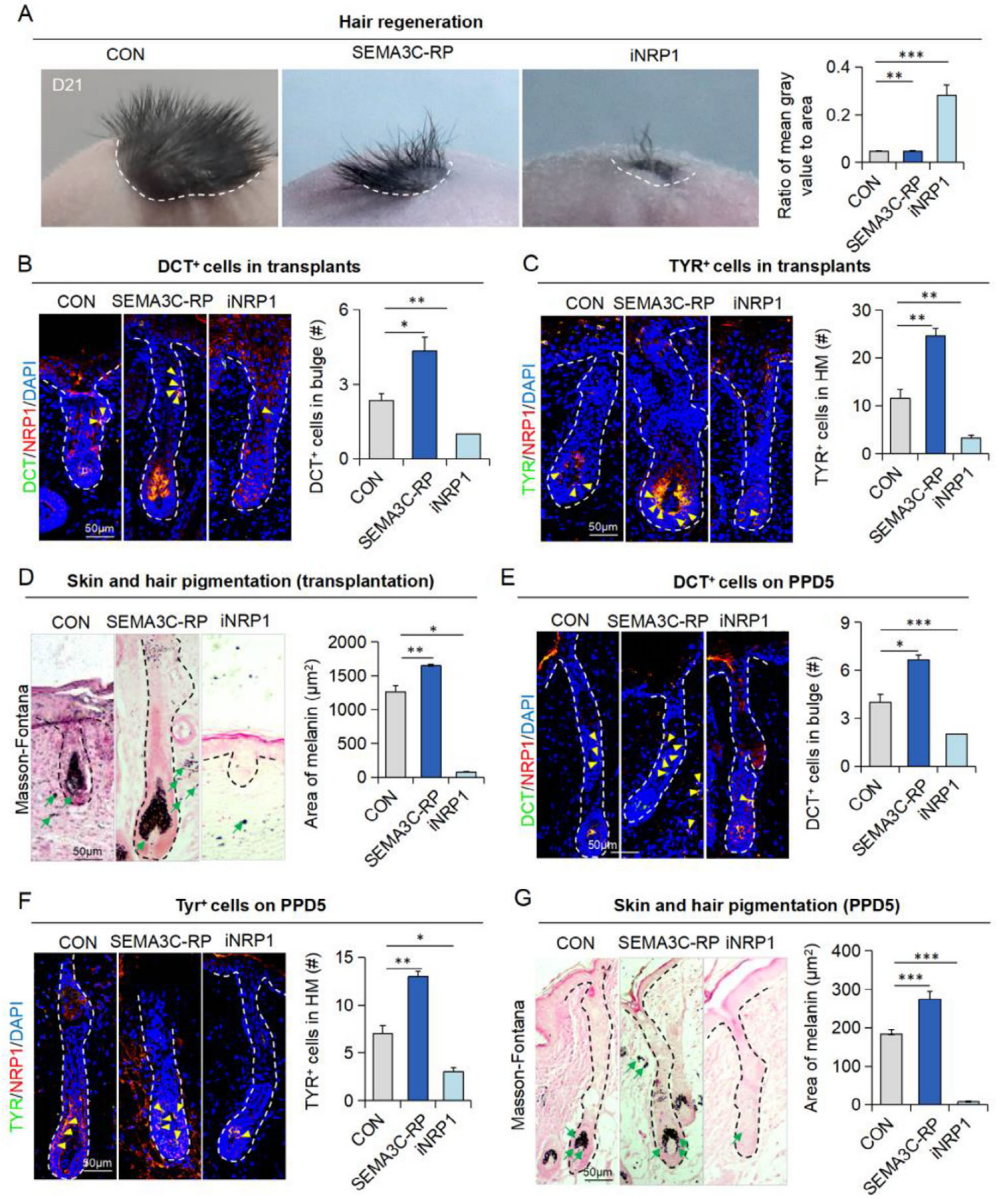
Functional characterization of the SEMA3C‐NRP1 axis in melanocytic pattern formation A) Transplanted skin organoids from the control group, SEMA3C‐activated group, and NRP1‐inhibited group at 21 days post‐transplantation. Quantification of the ratio of mean gray value to area. N ≥ 3; ^**^ *p* < 0.01, ^***^ *p* < 0.001. B) Immunofluorescence staining for DCT, NRP1, and DAPI, with quantification of DCT+ cells in skin organoids from the control, SEMA3C‐activated, and NRP1‐inhibited groups at 9 days post‐transplantation. N ≥ 3; ^*^ *p* < 0.05; ^**^ *p* < 0.01, scale bar = 50 µm. C) Immunofluorescence staining for TYR, NRP1, and DAPI, with quantification of TYR+ cells in the same groups. N ≥ 3; ^**^ *p* < 0.01, scale bar = 50 µm. D) Masson‐Fontana staining and quantification of melanin area in the same groups. N ≥ 3; ^*^ *p* < 0.05, ^**^ *p* < 0.01, scale bar = 50 µm. E) Immunofluorescence staining for DCT, NRP1, and DAPI, with quantification of DCT+ cells on Day 5 in plucked mice. N ≥ 3; ^*^ *p* < 0.05, ^***^ *p* < 0.001, scale bar = 50 µm. F) Immunofluorescence staining for TYR, NRP1, and DAPI, with quantification of TYR+ cells on Day 5 in plucked mice. N ≥ 3; ^*^ *p* < 0.05, ^**^ *p* < 0.01, scale bar = 50 µm. G) Masson‐Fontana staining and quantification of melanin area on Day 5 in plucked mice. N ≥ 3; ^***^ *p* < 0.001, scale bar = 50 µm.

Next, we investigated the role of SEMA3C‐NRP1 signaling in recruiting melanocytes to the Bulge region. To simulate this recruitment process, we transplanted skin organoids into nude mice and monitored hair growth and pigmentation at the graft site after 21 days. Treatment with SEMA3C recombinant protein (SEMA3C‐RP) resulted in hyperpigmentation of hair shafts, although hair regeneration was less robust compared to controls. Conversely, inhibition of NRP1 (iNRP1) blocked hair regeneration and reduced hair pigmentation (**Figure** **6**A). Immunostaining revealed that SEMA3C‐RP increased the number of melanocytes in both the Bulge and hair matrix, while Masson‐Fontana staining showed elevated melanin deposits in the hair matrix, likely contributing to the hyperpigmentation of grafted hair (Figure 6B–D). Notably, SEMA3C‐expressing cells were also increased in the Bulge region following SEMA3C‐RP treatment (Figure S6A, Supporting Information). In contrast, iNRP1 treatment nearly eliminated all melanocytes, leaving only minimal melanin deposits (Figure 6B–D).

Given that NRP1 inhibition impeded hair regeneration, we employed the hair‐plucking model for further validation. Consistently, SEMA3C‐RP enhanced melanocyte recruitment to the hair matrix and melanin deposition, effects that were inhibited by iNRP1 (Figure 6E–G; Figure S6B, Supporting Information). Collectively, our results demonstrate that SEMA3C released from Bulge cells regulates melanocyte recruitment and function via their surface receptor NRP1.

### 2.7 Tubb2b Functions Downstream of Nrp1

**Figure 7:**
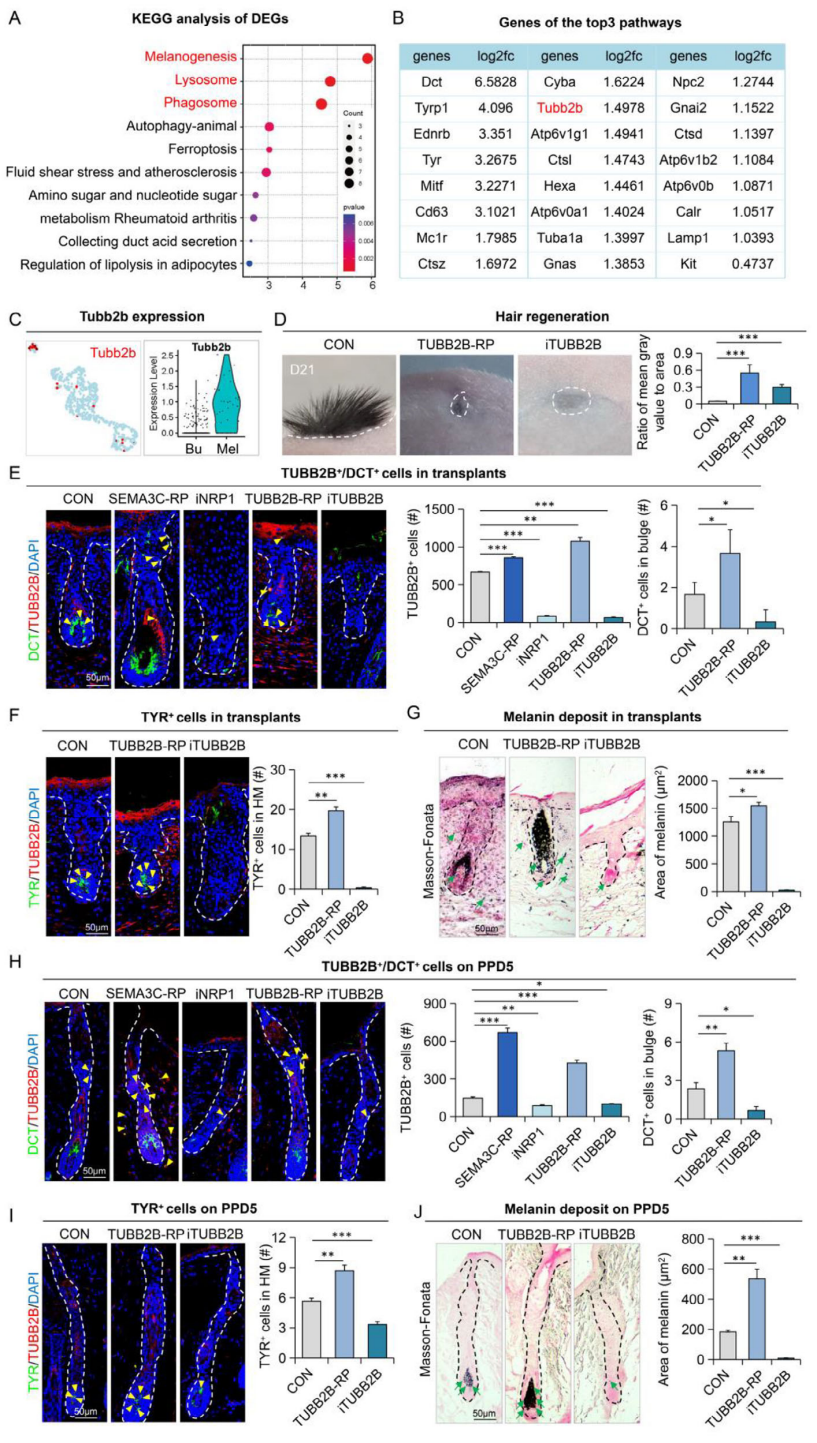
NRP1‐TUBB2B regulates melanocytic migration toward hair follicle bulge A) KEGG enrichment analysis of melanocytes showing differential pathways. B) Differentially expressed genes for the top three enriched pathways. C) Violin plots and feature plots of Tubb2b expression. D) Transplanted skin organoids from the control group, TUBB2B‐activated group, and TUBB2B‐inhibited group at 21 days post‐transplantation. Quantification of the ratio of mean gray value to area. N ≥ 3; ^***^ *p* < 0.001. E) Immunofluorescence staining for DCT, TUBB2B, and DAPI, with quantification of DCT+ and TUBB2B+ cells in skin organoids from the control, SEMA3C‐activated, NRP1‐inhibited, TUBB2B‐activated, and TUBB2B‐inhibited groups at 9 days post‐transplantation. N ≥ 3; ^*^ *p* < 0.05, ^**^ *p* < 0.01, ^***^ *p* < 0.001, scale bar = 50 µm. F) Immunofluorescence staining for TYR, TUBB2B, and DAPI, with quantification of TYR+ cells in the same groups. N ≥ 3; ^**^ *p* < 0.01, ^***^ *p* < 0.001, scale bar = 50 µm. G) Masson‐Fontana staining and quantification of melanin area in the same groups. N ≥ 3; ^*^ *p* < 0.05, ^***^ *p* < 0.001, scale bar = 50 µm. H) Immunofluorescence staining for DCT, TUBB2B, and DAPI, with quantification of DCT+ and TUBB2B+ cells on Day 5 in plucked mice. N ≥ 3; ^*^ *p* < 0.05, ^**^ *p* < 0.01, ^***^ *p* < 0.001, scale bar = 50 µm. I) Immunofluorescence staining for TYR, TUBB2B, and DAPI, with quantification of TYR+ cells on Day 5 in plucked mice. N ≥ 3; ^**^ *p* < 0.01, ^***^ *p* < 0.001, scale bar = 50 µm. J) Masson‐Fontana staining and quantification of melanin area on Day 5 in plucked mice. N ≥ 3; ^**^ *p* < 0.01, ^***^ *p* < 0.001, scale bar = 50 µm.

To elucidate the molecular mechanisms downstream of Nrp1, we performed KEGG enrichment analysis on differentially expressed genes (DEGs) in melanocytes. The top three enriched pathways identified were Melanogenesis, Lysosome, and Phagosome, with a comprehensive list of DEGs (**Figure** **7**A,B). Among all the DEGs involved in these top three pathways, Tubb2b was found to be highly expressed specifically in melanocytes (Figure 7C; Figure S7A,B, Supporting Information). Tubb2b is known to regulate microtubule stability, which is crucial for cell morphology, intracellular transport, and cell division.^[^ ^28^, ^29^, ^30^ ^]^ Again, we first used skin organoids to study the effect of the SEMA3C‐NRP1‐TUBB2B axis on melanocyte recruitment and function. Although both treatments with TUBB2B‐RP^[^ ^31^ ^]^ and iTUBB2B^[^ ^32^ ^]^ prevented hair regeneration (Figure 7D), we were able to identify several completely formed hair follicles in which we found increased melanocytes in the hair matrix and melanin deposition after TUBB2B‐RP treatment (Figure 7E–G). This was further verified in the hair plucking model where administration of TUBB2B‐RP indeed caused accumulation of melanocytes in both the Bulge and hair matrix, leading to hyper melanated hair matrix (Figure 7H–J). Once again, inhibition of TUBB2B resulted in a dramatic decrease in total melanocytes and a complete loss of melanin deposits in the hair matrix (Figure 7H–J). Taken together, these results clearly indicate that the adaptive patterning of melanocytes relies on β‐tubulin, which likely contributes to its migratory potential after receiving local stimuli such as SEMA3C.

**Figure 8:**
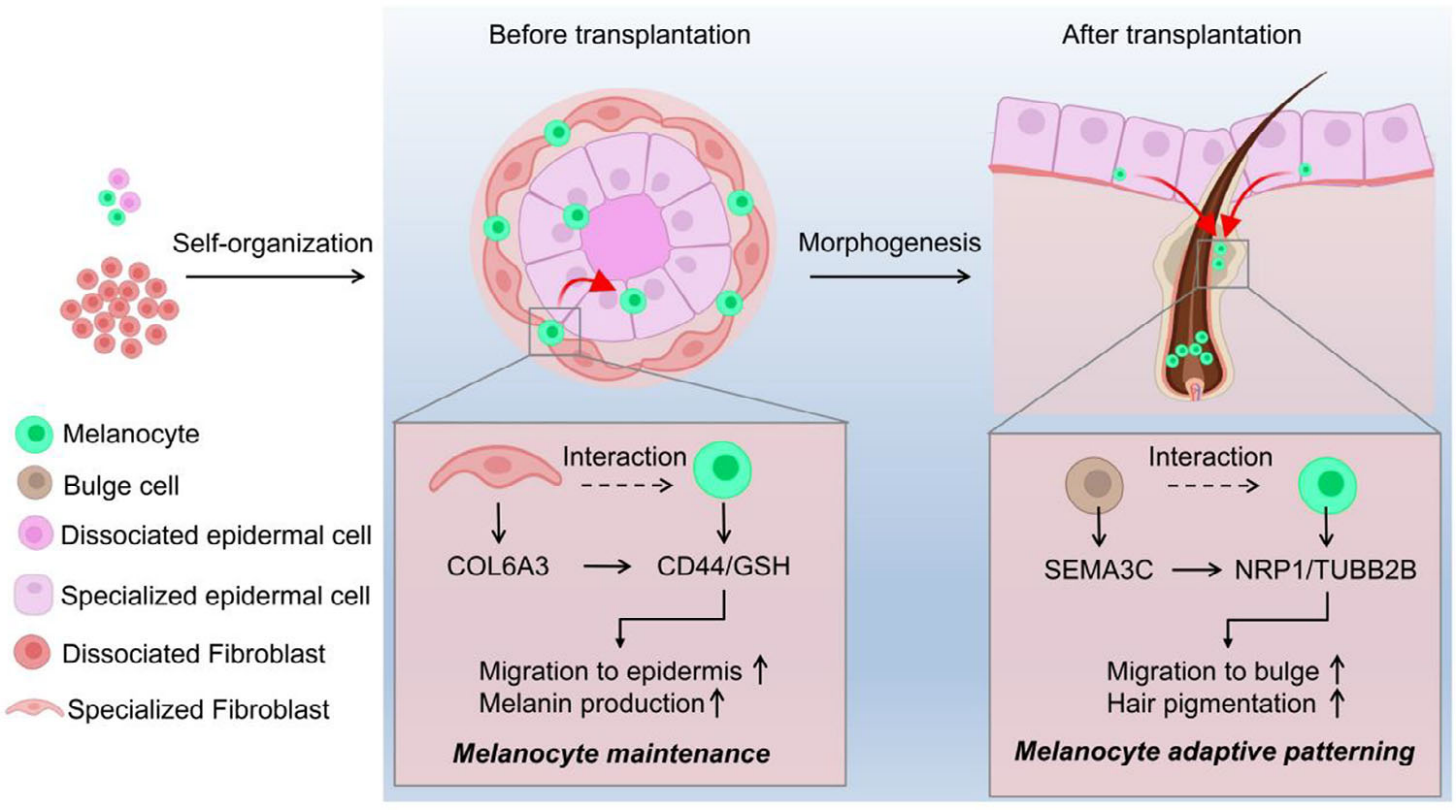
Graphical summary The release of COL6A3 by fibroblasts is sensed by the CD44 receptor on melanocytes, activating glutathione (GSH) metabolism and increasing melanocyte survival during skin organoid culture. SEMA3C released by bulge cells binds to the NRP1 receptor on melanocytes, regulating microtubule stability and promoting melanocyte recruitment and function during organoid‐based hair follicle neogenesis.

## 3 Discussion

To produce the vast variation of pelage colors and patterns, melanocytes must follow a set of strict preprogrammed genetic instructions yet be capable of adaptation in response to local biochemical instructions. The adaptive mechanism ensures that melanocytes can maintain stability and reactivity during development and in the dynamic changing tissue microenvironment, thus achieving precise regulation of pigmentation. However, the specific mechanisms have not been elucidated. Using our previously established skin organoid that undergoes in vitro self‐organization^[^ ^33^ ^]^ and in vivo morphogenesis to produce pigmented hair, we discover that a key two‐step mechanism that dictates the anatomical fate of melanocytes in the hair follicles simply lies within subtle changes of COL6A3 in the skin organoid culture system and SEMA3C in the hair follicle microenvironment. Our current study not only provides a molecular basis for variations of hair colors but also illuminates potential therapeutic strategies for hair pigmentation disorders (**Figure** **8**).

Our findings from mouse skin organoids suggest that the autonomous organization and adaptive patterning of melanocytes after dissociation may involve an “architectural memory” that allows them to respond to identical biological cues and maintain specific behavioral patterns in the absence of external stimuli. While preservation of epigenetic changes has been proposed to explain rapid alteration of gene expression or adjustment of signal transduction pathways following microenvironmental changes,^[^ ^34^, ^35^ ^]^ our results suggest that the core driver of melanocyte patterning is dependent on local instructions given by dermal fibroblasts through COL6A3 recognized by melanocytic CD44 receptors. This can be deduced by the seemingly unpatterned distribution of melanocytes in the self‐organized cyst‐like structures prior to transplantation and migration toward the epidermis and then into the developing hair follicle after transplantation, mirroring their behaviors during embryonic development. Most importantly, this mechanism maintains a healthy population of melanocytes in the skin organoid culture, which subsequently allows them to respond to local molecular instructions after transplantation, thus forming the first step of the two‐step mechanism. During the early stages of hair follicle formation, the Wnt/β‐catenin pathway plays a pivotal role, contributing to the specification of melanocyte lineages and promoting the proliferation and differentiation of melanocyte stem cells (McSC).^[^ ^36^ ^]^ As hair follicles develop, the Notch pathway begins to influence cell fate decisions, maintaining a delicate balance between McSC self‐renewal and differentiation.^[^ ^37^ ^]^ This balance is essential for the cyclical nature of hair pigmentation, ensuring that only a portion of the progenitor cells differentiate into mature melanocytes while preserving the McSCs reservoir for future hair follicle regeneration cycles. Interestingly, while dermal fibroblast‐derived COL6A is mostly absent in the hair follicle, it can be distinctively detected in the papillary dermis region. Thus, we speculate that this restricted expression of COL6A3 may also act both as a stemness maintainer and a “pruning” mechanism resulting in differentiated melanocytes present only in the skin organoids. That is, those melanocytes that are unable to sense the COL6A signal may undergo programmed cell death. Indeed, COL6A3 has been identified as a key gene involved in the PI3K pathway that promotes melanocyte proliferation and differentiation, which corroborates our observation that both DCT‐and TYR‐expressing melanocytes were enriched particularly in the hair bulb.^[^ ^38^ ^]^

Unexpectedly, our data further suggest that this selective pressure is linked to GSH metabolism as Col6a3‐mediated activation of Cd44 in melanocytes increased several genes that play pivotal roles in this process, such as Gsta4. Gsta4 encodes Glutathione S‐transferase 4, which catalyzes the conjugation of reduced GSH to 4‐hydroxynenal or other reactive carbonyl byproducts of peroxidative degradation.^[^ ^39^ ^]^ This is critical for hair pigmentation as elevated oxidative stress impedes the generation of melanosomes.^[^ ^40^ ^]^ Thus, we suspect that up‐regulation of Gast4 is more than just an indication of active melanosome production, it also serves a protective role in maintaining melanocyte population. However, when GSH levels become elevated, as shown in our study and others, such as due to its tyrosinase inhibitory function, melanocytes lose their ability to pigment hair shafts.^[^ ^41^, ^42^, ^43^ ^]^ Moreover, through this intricate balancing of GSH levels, melanocytes undergo a selection process where prolonged exposure to COL6A3 and activation of their CD44 surface receptors are required for their longevity and survival at the pre‐determined anatomical site in the hair follicle, thus contributing to their adaptive patterning during hair follicle morphogenesis.

Another intriguing discovery of our study is that there exists a reciprocal interaction between the bulge cells and melanocytes in the developed hair follicle, which forms the second‐step of the two‐step mechanism. Unlike other stem cells, melanocyte stem cells are highly plastic in cell state transition.^[^ ^44^ ^]^ While it has been reported in recent years that mature melanocytes are capable of dedifferentiation and migrating back to their niche in the bulge to repopulate their progenitors in a cyclic manner, it is not fully known the local cues that trigger such migratory motion.^[^ ^45^ ^]^ Here we newly identified that the recruitment of melanocytes to the Bulge region is governed by SEMA3C‐NRP1 signaling between bulge cells and melanocytes, respectively. This pathway also instructs melanocytes to migrate to the hair matrix in the hair bulb, leading to increased hair regeneration and pigmentation. This mechanism of adaptive patterning is achieved through activation of β‐tubulin, the chief cytoskeletal protein controlling cell division, organelle localization, intracellular transport, and cell migration.^[^ ^46^ ^]^ Activation of β‐tubulin and dynamic regulation of microtubules in Xenopus oocytes can trigger their self‐patterning into cell‐like units at regular intervals. When the microtubule stabilizing drug paclitaxel is added to the extract of mitotic xenopus eggs, rapid self‐patterning to form the radiating array can be observed.^[^ ^47^ ^]^ Nevertheless, it has been reported that increasing microtubule stability can also promote the migratory ability of melanoma cells, and further promote the enhancement of the aggressiveness of melanoma cells.^[^ ^48^ ^]^ These results align with our findings that the SEMA3C‐NRP1‐TUBB2B axis allows adaptive patterning of melanocytes by promoting microtubule stabilization and thus enhancing the recruitment of melanocytes to the bulge for niche population maintenance and hair matrix for hair pigmentation. Notably, inhibition of both COL6A3‐CD44 and SEMA3C‐NRP1‐TUBB2B led to a significant reduction of both hair pigmentation and hair content. Studies have shown that melanocytes are not only key cells in pigmentation but also affect the activity of hair follicle stem cells by secreting signaling molecules, such as Osteopontin.^[^ ^49^ ^]^ Osteopontin is a major signaling factor secreted by nevus melanocytes, which can activate hair follicle stem cells through interaction with CD44 receptors, thereby promoting hair renewal.^[^ ^49^ ^]^ This suggests that the function of melanocytes not only directly influences hair pigmentation, but also indirectly influences hair regeneration by regulating the activity of hair follicle stem cells.

Given the central roles of COL6A3–CD44 and SEMA3C–NRP1 signaling in melanocyte regulation and hair pigmentation, our findings highlight promising translational avenues. Although no specific drugs currently target these pathways, their therapeutic potential is supported by existing clinical applications. For example, collagens like COL6A3 are already used in dermatology to improve skin elasticity, and COL17A1^[^ ^50^ ^]^ supplementation has shown efficacy in delaying hair follicle aging, suggesting that COL6A3 may similarly enhance pigmentation and reduce greying. Meanwhile, the SEMA3C–NRP1 axis, which governs melanocyte migration and activity, could be targeted using monoclonal antibodies or small molecules. Advances in blocking NRP1 interactions, particularly in the VEGF pathway, demonstrate the druggability of this receptor.^[^ ^51^ ^]^ Together, these insights offer a structural and pharmacological basis for developing novel therapies aimed at restoring pigmentation in disorders such as vitiligo or post‐inflammatory hypopigmentation, where reactivating melanocyte function is essential for effective re‐pigmentation.

However, this model has limitations in simulating the complex physiological environment of human beings. Compared with the skin organoids derived from human iPSCs, though the neonatal mouse model is easy to operate and has a lower cost, it lacks the complex structure and long‐cycle growth characteristics of human hair follicles and cannot fully simulate the in vivo circulation and immune response. This may lead to an overestimation of melanocyte function and pigmentation mechanisms, especially in terms of long‐term dynamic balance and systemic regulation. Future research needs to construct models that are closer to the human physiological environment, such as humanized organoids with immune co‐culture systems, to simulate the long‐term regulation and pathological microenvironment of hair follicles. Meanwhile, through cross‐species single‐cell transcriptome comparison, the conservation of the COL6A3‐CD44 pathway in maintaining the stemness of human melanocytes was analyzed, and a microfluidic organoid system was developed to simulate the vascularized microenvironment, thereby accurately evaluating the interaction between systemic and local signals.

In conclusion, the present study has revealed a previously unrecognized dual mechanism that orchestrates melanocyte fate during hair follicle development and regeneration. We demonstrate that the early COL6A3–CD44–GSH axis maintains melanocyte populations by mitigating oxidative stress, while the later SEMA3C–NRP1–TUBB2B pathway reciprocally guides melanocytes back to the hair follicle niche to enable pigmentation and tissue patterning. Although these pathways appear functionally distinct, disruption of either results in a significant loss of melanocytes, indicating their mutual reliance. We propose that these mechanisms cooperatively regulate melanocyte behavior during follicle development. These findings not only deepen our understanding of melanocyte biology but also open new avenues for therapeutic intervention. By targeting the COL6A3–CD44 and SEMA3C–NRP1 pathways, this work lays a compelling theoretical foundation for developing innovative treatments for pigmentation disorders such as vitiligo.

## 4 Experimental Section

### Mice

CD1, C57BL/6, and nude mice were purchased from Vital River (Beijing, China). The mice were housed in a temperature‐controlled facility maintained at 25°C with a 12 h light/dark cycle. To ensure adequate living space and minimize stress, no more than 3–5 mice were kept per cage. All animals had ad libitum access to a standard rodent diet and clean drinking water to meet their nutritional and physiological needs. All experimental procedures were conducted in strict accordance with institutional and national ethical guidelines for animal research and were approved by The First Hospital of China Medical University (Approval No. AF‐SOP‐07‐1.2‐01).

### Skin Organoid Culture

The skin organoids were generated as described before.^[^ ^12^ ^]^ Briefly, the dorsal skin of the neonatal mice was resected and spread evenly in a petri dish. It was treated with 0.25% trypsin to separate the epidermis from the dermis. Subsequently, the epidermis and dermis were digested with 0.35% type I collagenase (#LS004197, Worthington, USA), respectively, to obtain dissociated cells. During this process, melanocytes within the dermis were digested into single cells. Subsequently, the epidermal cells and dermal cells were mixed in a ratio of 1:9, and they were centrifuged and resuspended. Then, a total number of 1× 10^7^ cells were carefully transferred to each of the Transwell inserts in a 12‐well plate and settled by gravity. Finally, 700 µL of DMEM/F12 medium (#MT10013CV, Corning, USA) containing 10% fetal bovine serum (#10099‐141C, Gibco, USA) was added along the well wall and incubated in a 37°C, 5% CO₂ incubator. The day of seeding was designated as Day 0, and samples were collected at 6 h, Day 2, Day 4, and Day 7 for the subsequent analyses. The culture medium was replaced every two days.

### Immunofluorescence Staining

Skin samples were collected from embryonic mice at embryonic days (E) 12.5, 13.5, and 14.5. The pregnant mice were deeply anesthetized prior to surgery, after which the embryos were carefully retrieved via hysterotomy, and then dorsal skin was removed surgically. Organoid and tissue samples were fixed in 4% PFA (paraformaldehyde) at 4°C, paraffin‐embedded, and cut into 6‐or 8‐micron sections, respectively. Before staining, the sections were dewaxed, hydrated, and antigen‐repaired with citric acid (#C805019, Macklin, China) solution. The samples were then blocked with 2% bovine serum albumin (BSA; #A8020, Solarbio, China) for 1 h at 37°C followed by incubation with primary antibodies overnight at 4°C and fluorochrome‐conjugated secondary antibodies at 37°C for 2 h. Samples were counterstained by DAPI for 30 min at room temperature. Lastly, the samples were sealed in an anti‐fluorescence quencher with a cover glass using nail polish. Images were taken under a confocal microscope (Leica, Germany) at the Analysis and Testing Center of Chongqing University. Primary and secondary antibodies are shown in Table S1 (Supporting Information). The immunofluorescence images were quantified by ImageJ software version 1.8.0 with the help of the cell counter add‐on function.

### Masson‐Fontana Staining

The samples were dewaxed and rehydrated as described previously. To visualize melanin deposition, the samples were stained using a Masson‐Fontana Staining Kit (Solarbio, China) according to the manufacturer's instructions. After staining, the samples were dehydrated and sealed with neutral resin for observation under a light microscope. The images were subsequently analyzed by ImageJ for the area of melanin deposit.

### Western Blotting

Cells were washed with cold PBS, and total proteins were extracted using lysis buffer supplemented with protease inhibitors. Lysates were incubated on ice for 30 min and centrifuged at 12 000 × g for 15 min at 4 °C, after which the supernatant was collected. Protein concentrations were determined using the BCA assay (Thermo Fisher Scientific). Equal amounts of protein were mixed with SDS loading buffer, denatured by boiling for 5 min and separated by a 10% SDS‐PAGE gel. Electrophoresis was carried out at 120 V and the proteins were transferred onto a PVDF membrane (Millipore). Membranes were blocked with 5% non‐fat milk in TBST for 1 h at room temperature, incubated with primary antibodies overnight at 4 °C, and then with HRP‐conjugated secondary antibodies for 1 h at room temperature. After thorough washing with TBST, protein bands were detected using an enhanced chemiluminescence (ECL) system (Bio‐Rad).

### Functional Assays using Small Molecule Drugs and Recombinant Proteins

The dorsal hair of 8‐week‐old C57BL/6 mice was plucked in the direction of hair growth using wax as described before.^[^ ^17^ ^]^ The day of hair removal was denoted as post‐plucking day 0 (PPD0). To conduct functional assays in vitro, small molecule drugs or recombinant proteins were added to the culture medium of the organoids at various optimized concentrations prior to the analyses. For in vivo functional assays, the small‐molecule drugs or recombinant proteins were injected subcutaneously into the dorsal skin. The injection was repeated on PPD2 and 4, and the skin samples around the injection site were collected on PPD5. The names, catalog numbers, concentrations, and injection volumes of the small molecule drugs and recombinant proteins are listed in Table S2 (Supporting Information).

### Transplantation

The skin organoids were grafted to the dorsal skin of nude mice as previously described.^[^ ^12^ ^]^ In brief, a circular wound area ≈1 cm in diameter was created on the back of nude mice. The 4‐day‐old skin organoids on the Transwell insert were removed along with the membrane which was flipped onto the wound area of the host with the cell side down. The wound area was bandaged to protect the wound and facilitate healing. After 3 weeks, the bandages were removed, and the regenerated hair was photographed and quantified using ImageJ.

### ScRNA‐Seq Analysis

The scRNA‐seq (Single cell RNA sequence) data of mouse skin organoids^[^ ^18^ ^]^ (GSE215980) were obtained from the GEO (Gene Expression Omnibus) database. Quality control on the raw data was performed to remove low‐quality cells and genes, such as filtering out cells with a total UMI count of less than 200 or more than 7500 and removing cells with more than 20% mitochondrial genes. The Seurat package was then used for data normalization and principal component analysis (PCA) for dimensionality reduction. The Leiden algorithm was used for cluster analysis to identify different cell subpopulations. The clustering parameter is set to a resolution of 0.3 to ensure the rationality of the clustering results.^[^ ^19^ ^]^ Seurat v.4.3.0. was used to perform QC, normalization, feature selection, linear and nonlinear dimension reduction, cell clustering using biomarkers, and cell type identification assignment. Cells with a mitochondrial content greater than 5% were excluded and a dimensionality reduction by UMAP (Uniform Manifold Approximation and Projection) was performed for dimensionality reduction. CellChat was used to analyze the interactions between melanocytes and other cell groups. KEGG (Kyoto Encyclopedia of Genes and Genomes) enrichment analyses were performed and visualized using an online platform (https://www.bioinformatics.com.cn).

### Protein Docking

Crystal structures of COL6A3 (PDB#: 4IHK), CD44 (PDB#: 4MRF), NRP1 (PDB#: 6FMC), and PLXNA4 (PDB#: 4E74) were retrieved from the Protein Data Bank (PBD) (https://www.rcsb.org). The protein structure of SEMA3C was predicted using AlphaFold3 (https://alphafold.com). AlphaFold3 was used to perform protein‐protein docking of the above receptor‐ligand pairs. Subsequently, the Z‐score was calculated by comparing the experimentally observed interaction energy with the predicted random interaction energy through the DockEasy online tool (https://www.dockeasy.cn) to evaluate the accuracy and reliability of the prediction model for the complex structure.

### Statistical Analyses

All data were presented as the mean ± standard deviation (SD). The samples were compared using either unpaired two‐tailed Student's *t*‐tests or one‐way analysis of variance (ANOVA) followed by Dunnett's *post hoc* tests. P values < 0.05 were considered statistically significant. The exact number of biological replicates (n) of each experiment performed was displayed on the graphs as individual data points and described in the figure legends.

### Ethical Statement

The experimental protocols were approved by The First Hospital of China Medical University (Approval No. AF‐SOP‐07‐1.2‐01).

## Conflict of Interest

The authors declare no conflict of interest.

## Author Contributions

T.L. performed conceptualization, methodology, data curation, investigation, wrote the final manuscript. X.L performed validation, visualization, data curation. X.X. wrote, reviewed, and edited the final manuscript. J.H., X.S., M.W., J.J., S.S., Z.L., T.X., D.L., Y.Z., R.M., X.X. performed methodology and visualization. W.S., J.L. performed supervision and funding acquisition. M.L., W.W., C.‐M.C. performed conceptualization, supervision, project administration, funding acquisition, and wrote, reviewed, and edited the final manuscript.

## Supporting information



Supporting Information

## Data Availability

The data that support the findings of this study are available from the corresponding author upon reasonable request.
