# Supplementary material for: Regenerative Hair Pigmentation via Skin Organoids: Adaptive Patterning Mediated by Collagen VI and Semaphorin 3C
Source: Adv Sci (Weinh). 2025 Jul 3;12(36):e02436. doi: 10.1002/advs.202502436 (PMC12462959; doi:10.1002/advs.202502436)
Supplement: Supplementary file 1 — Supporting Information [file ADVS-12-e02436-s001.docx]

**SUPPLEMENTARY MATERIALS**

**Table S1. Antibodies used in this study.**

| **Antibody** | **Ratio** | **Company** | **Cat #** | **Species** |
| --- | --- | --- | --- | --- |
| Vimentin | 1:300 | Beyotime | AF0318 | Mouse |
| E-cadherin | 1:200 | Beyotime | AF0138 | Mouse |
| Krt14 | 1:500 | Boster | A01432 | Rabbit |
| Dct | 1:500 | Abcam | ab74073 | Rabbit |
| Tyr | 1:500 | Abcam | ab170905 | Rabbit |
| Gsta4 | 1:200 | Affinity | DF12203 | Rabbit |
| Sema3c | 1:200 | Affinity | DF8610 | Rabbit |
| Nrp1 | 1:100 | R&D | AF566-SP | Goat |
| Tubb2b | 1:200 | Novus | NBP2-46250 | Rabbit |
| Sox9 | 1:500 | Thermo | 14-9765-80 | Mouse |
| Collagen Ⅵ | 1:200 | Beyotime | AF2740 | Rabbit |
| Cd44 | 1:200 | Beyotime | AF0105 | Rabbit |

**Table S2. Small molecule inhibitors and activators used in this study.**

| **Cat #** | **Product description** | **Concentration and amount used** |
| --- | --- | --- |
| HY-P75684 | COL6A3 Protein | Skin organoid: 20 ng/mL; mice: 200 ng/mL (50 μL) |
| HY-P71282 | SEMA3C Protein | Skin organoid: 20 ng/mL; mice: 200 ng/m (50 μL) |
| HY-B0015 | Paclitaxel | Skin organoid: 10 μM; mice: 10 mM (50 μL) |
| HY-D0187 | L-Glutathione reduced | Skin organoid: 50 μM; mice: 50 mM (50 μL) |
| HY-10799 | EG00229 trifluoroacetate | Skin organoid: 10 μM; mice: 10 mM (50 μL) |
| HY-13520 | Nocodazole | Skin organoid: 10 μM; mice: 10 mM (50 μL) |
| HY-P2230 | Angstrom6 | Skin organoid: 50 μM; mice: 50 mM (50 μL) |
| LS004196 | Collagenase, Type 1 | Skin organoid: 20 ng/mL |
| HY-P70528 | SCF Protein, Mouse | Skin organoid: 20 ng/mL |

**SUPPLEMENTARY FIGURES and LEGENDS**

**
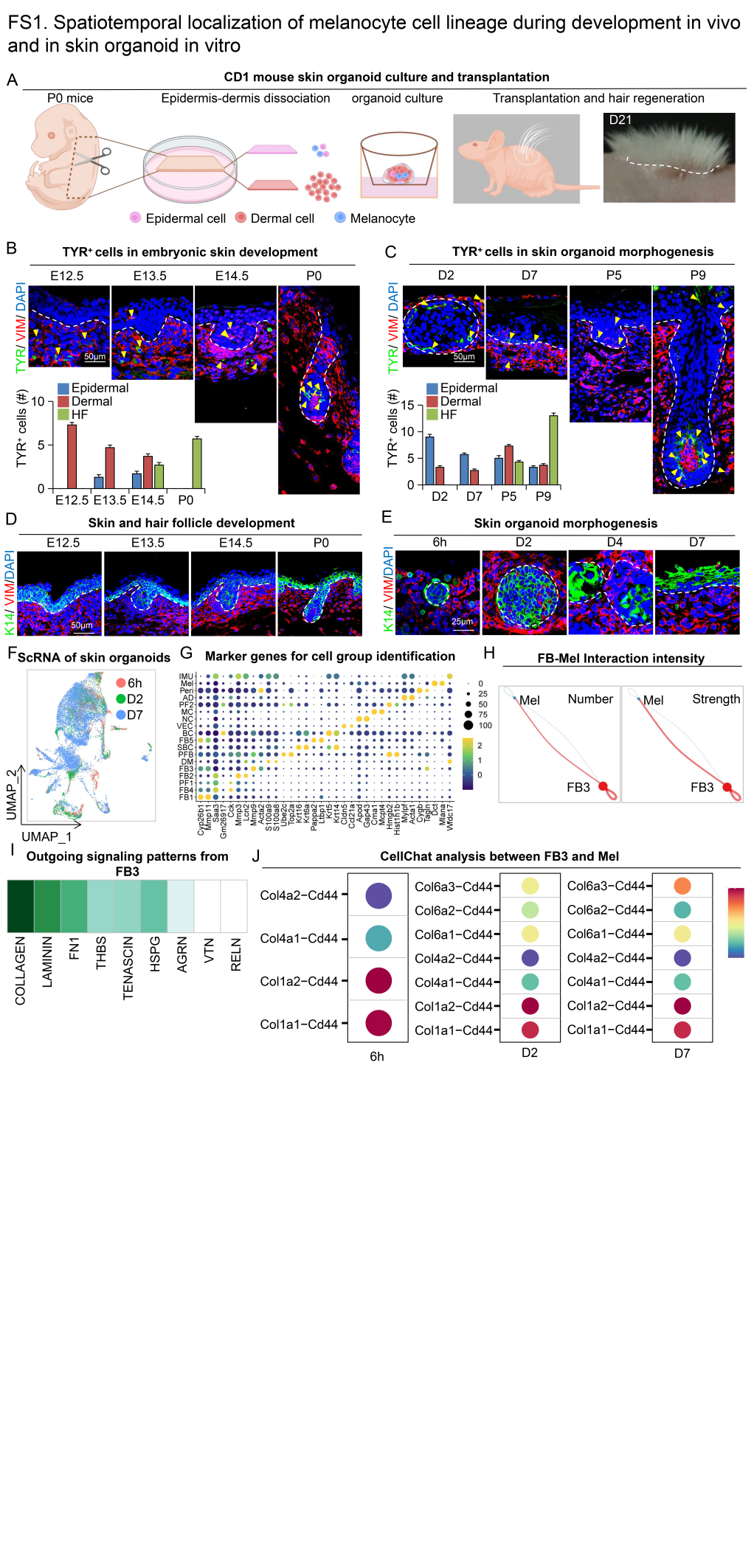
**

**Figure S1. Localization and dynamics of melanocyte lineage in skin organoids during early development**
A. Schematic representation of in vitro culture and in vivo transplantation of CD1 mouse skin organoids.
B. Immunofluorescence staining and quantification for TYR, VIMENTIN, and DAPI, and graphical illustrations showing the localization of melanocytes in embryonic skin at E12.5, E13.5, E14.5, and neonatal stages. N ≥ 3, scale bar = 50 μm.
C. Same as in B but during CD1 mouse skin organoid morphogenesis. N ≥ 3, scale bar = 50 μm.
D. Immunofluorescence staining for KRT14, VIMENTIN, and DAPI in embryonic skin at E12.5, E13.5, E14.5, and neonatal stages. Scale bar = 50 μm.
E. Immunofluorescence staining of KRT14, VIMENTIN, and DAPI in mouse skin organoids cultured in vitro for 6 hours, Day 2, Day 4, and Day 7. Scale bar = 25 μm.
F. Integrated clustering analysis of single-cell data from mouse skin organoids at 6 hours, Day 2, and Day 7.
G. Dot plot showing marker genes for each cell cluster.
H. Interaction number and intensity of the FB3-melanocyte signaling network.
I. Heatmap showing cell interaction pathways involving FB3.
J. Interactions between FB3 and melanocytes in the collagen pathway across different time points.

**
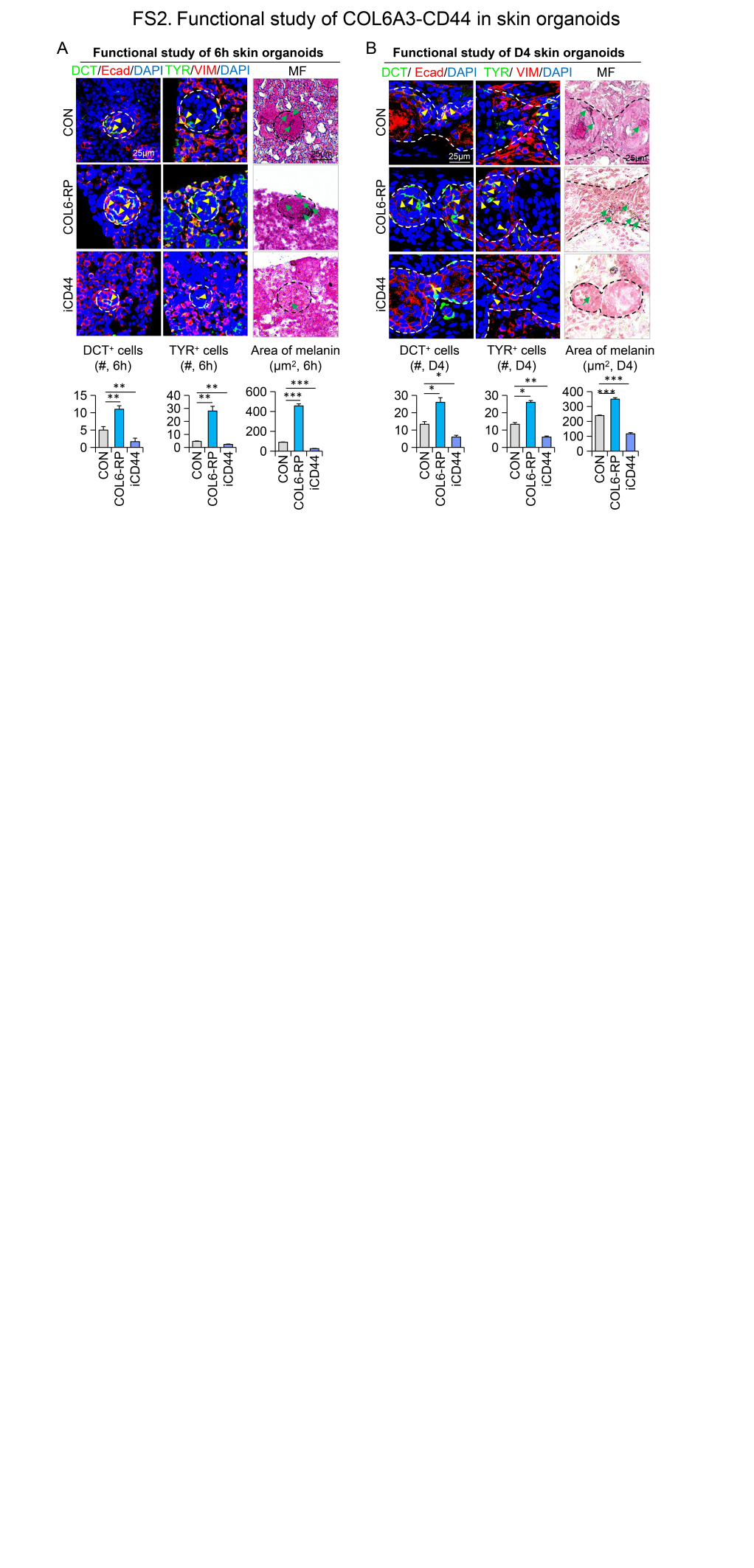
**

**Figure S2. Functional study of the COL6A3-CD44 axis in skin organoids**
A. Masson-Fontana staining and immunofluorescence staining for DCT, E-cadherin, DAPI, TYR, and VIMENTIN in 6-hour skin organoids from the control, COL6A3-treated, and CD44-inhibited groups. Quantifications show DCT+ or TYR+ cells and melanin area. N ≥ 3; **p < 0.01, ***p < 0.001; scale bar = 25 μm.

B. Same as in A. but in Day 4 skin organoids. N ≥ 3; *p < 0.05, **p < 0.01, ***p < 0.001; scale bars = 25 μm.

**
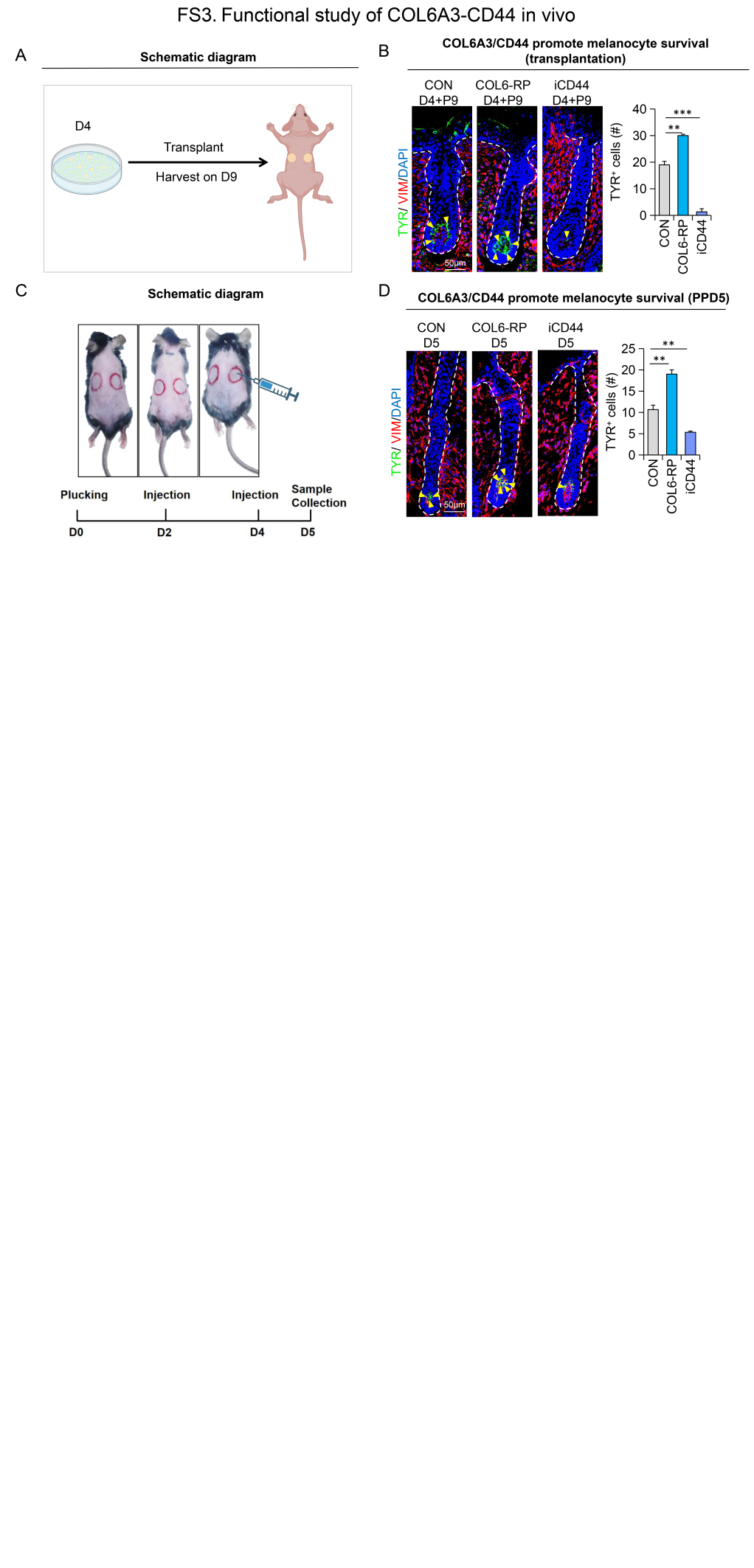
**

**Figure S3. Functional study of the COL6A3-CD44 axis in vivo**
A. Schematic representation of skin organoid transplantation into nude mice.
B. Immunofluorescence staining for TYR, VIMENTIN, and DAPI, with quantification of TYR+ cells in skin organoids at 9 days post-transplantation. N ≥ 3; **p < 0.01, ***p < 0.001; scale bar = 50 μm.
C. Schematic of the functional study in plucked mice.
D. Immunofluorescence staining for TYR, VIMENTIN, and DAPI and quantification of TYR+ cells in skin organoids on Day 5 in plucked mice. N ≥ 3; **p < 0.01; scale bar = 50 μm.

**
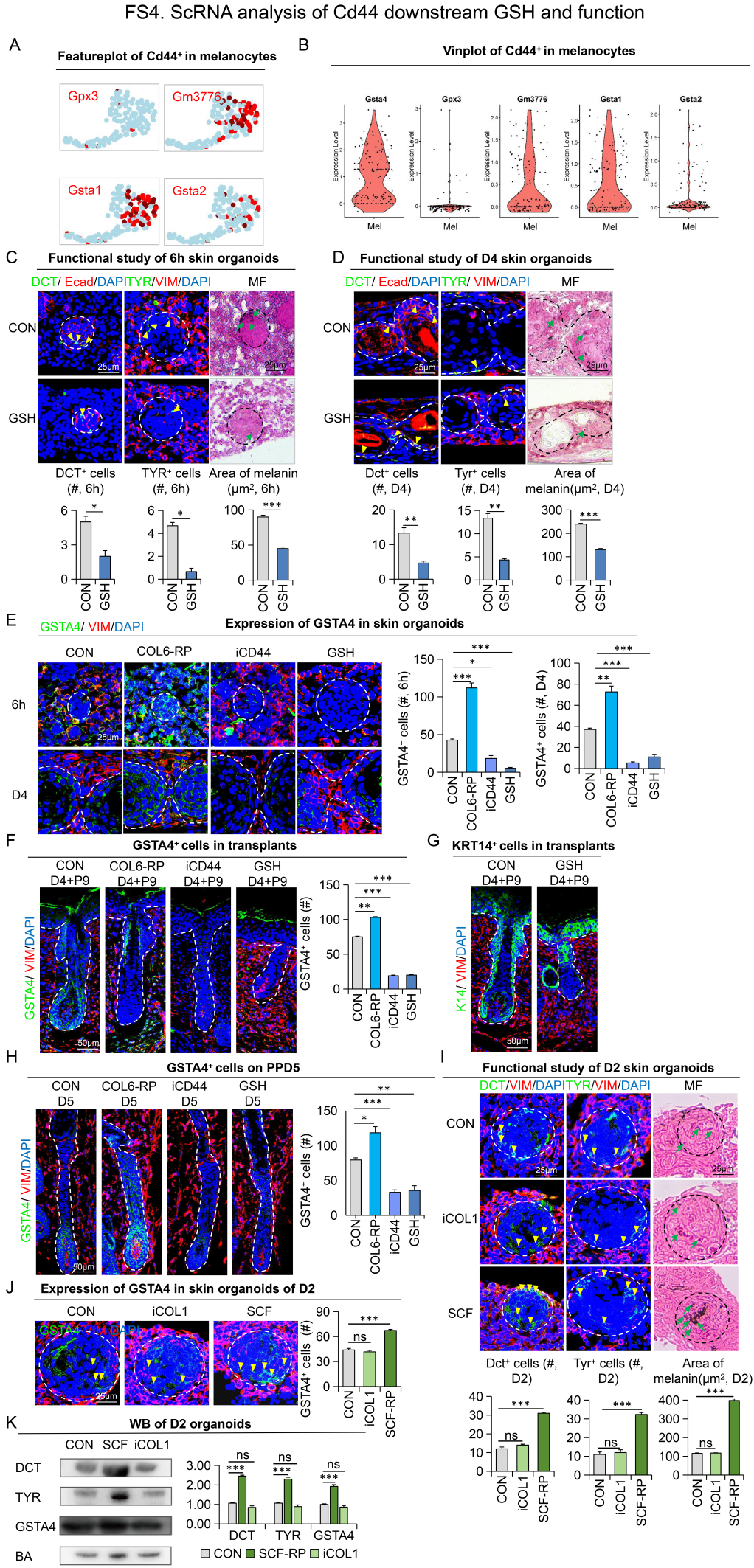
**

**Figure S4. scRNA analysis of CD44 downstream glutathione pathway and its function.**
A. UMAP plots showing Gpx3, Gm3776, Gata1, and Gata2 expression in the melanocyte cluster by unbiased clustering.
B. Violin plots showing the expression of the same genes as in A.
C. Masson-Fontana staining and immunofluorescence staining for DCT, E-cadherin, DAPI, TYR, and VIMENTIN in 6-hour skin organoids from the control and glutathione addition groups. Quantification of melanin area, DCT+ cell count, and TYR+ cell count. N ≥ 3; *p < 0.05, ***p < 0.001, scale bar = 25 μm.
D. Masson-Fontana staining and immunofluorescence staining for DCT, E-cadherin, DAPI, TYR, and VIMENTIN in Day 4 skin organoids from the control and glutathione addition groups. Quantification of melanin area, DCT+ cell count, and TYR+ cell count. N ≥ 3; **p < 0.01, ***p < 0.001, scale bar = 25 μm.
E. Immunofluorescence staining and statistics of GSTA4 in skin organoids under control, COL6A3 activation, CD44 inhibition, and glutathione treatment at 6h and Day 4. N ≥ 3; *p < 0.05, **p < 0.01, ***p < 0.001, scale bar = 25 μm.
F. Immunofluorescence staining for GSTA4 with quantification of GSTA4+ cells in the control group, COL6A3-activated group, CD44-inhibited group, and glutathione addition group at 9 days post-transplantation. N ≥ 3; **p < 0.01, ***p < 0.001, scale bar = 25 μm.
G. Immunofluorescence staining for GSTA4 with quantification of GSTA4+ cells in the control group, COL6A3-activated group, CD44-inhibited group, and glutathione addition group on Day 5 in plucked mice. N ≥ 3; *p < 0.05, **p < 0.01, ***p < 0.001, scale bar = 50 μm.
H. Immunofluorescence staining for KRT14 in skin organoids from the control and glutathione addition groups at 9 days post-transplantation. Scale bar = 50 μm.

1. Masson-Fontana staining and immunofluorescence staining for DCT, VIMENTIN , DAPI, TYR, and VIMENTIN in Day 2 skin organoids from the control group, SCF-activated group, COL1-inhibited group. Quantification of melanin area, DCT+ cell count, and TYR+ cell count. N ≥ 3; **p < 0.01, ***p < 0.001, scale bar = 25 μm.
2. Immunofluorescence staining and statistics of GSTA4 in skin organoids under control, SCF activation, COL1 inhibition treatment at Day 2. N ≥ 3; *p < 0.05, **p < 0.01, ***p < 0.001, scale bar = 25 μm.
3. Representative Western blot images and quantifications show up-regulation of DCT, TYR, and GSTA by SCF-RP and no significant difference of these proteins after treatment with iCOL1 in D2 mouse skin organoids. The results are shown as mean ± S.D., and were analyzed by One-way ANOVA with Dunnett’s post-hoc test. *p < 0.05, **p < 0.01, and ***p < 0.001. N=3.

**
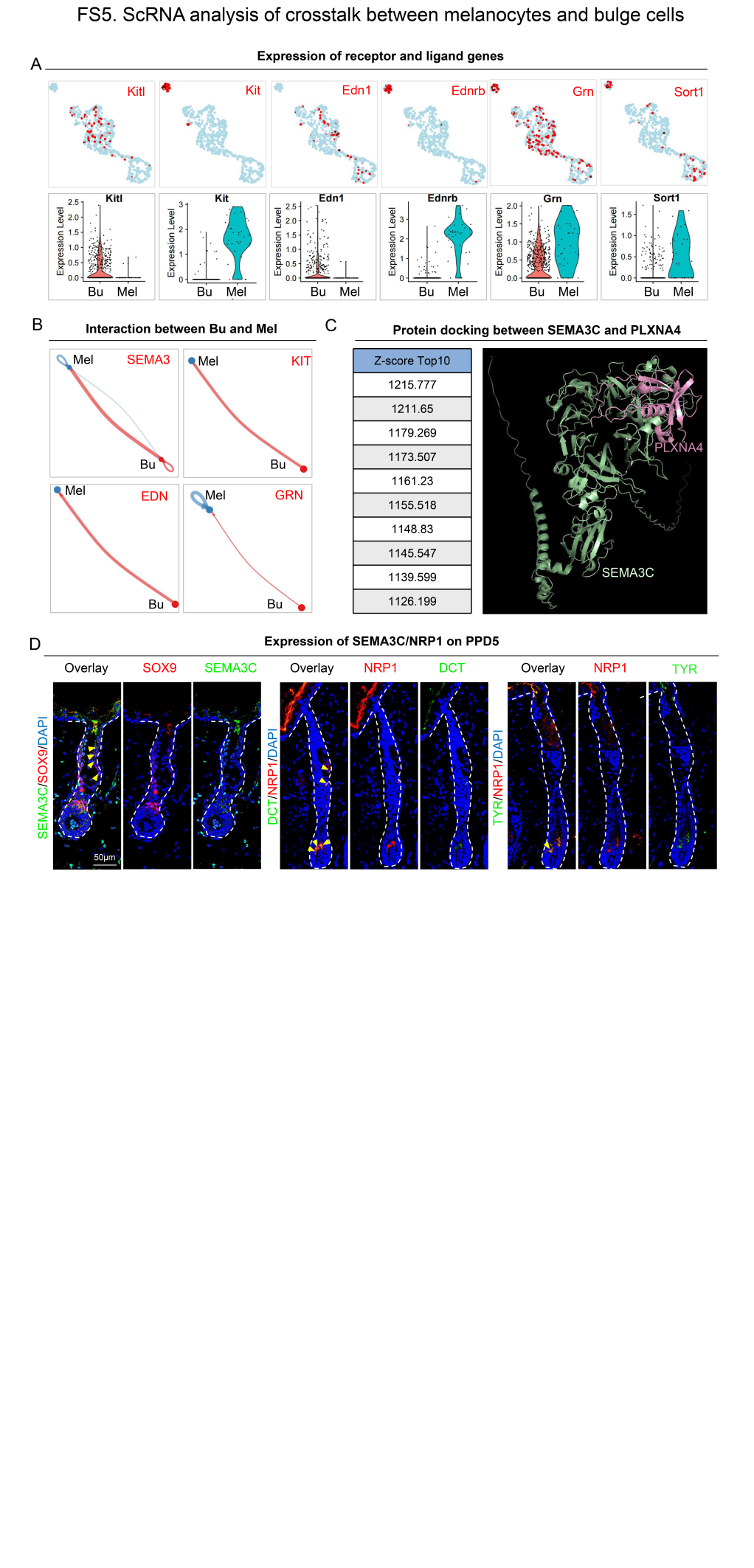
**

**Figure S5. scRNA analysis reveals crosstalk between melanocytes and bulge cells**
A. Violin plots and feature plots of Kitl, Kit, Edn1, Ednrb, Grn, and Sort1 expression.
B. Network diagram showing interaction pathways between bulge cells and melanocytes.
C. Protein docking analysis of the interaction between SEMA3C and PLXNA4.
D. Immunofluorescence for SEMA3C, SOX9, DAPI, DCT, NRP1, and TYR on day 5 post-pluck in mice. Scale bar = 50 μm.

**
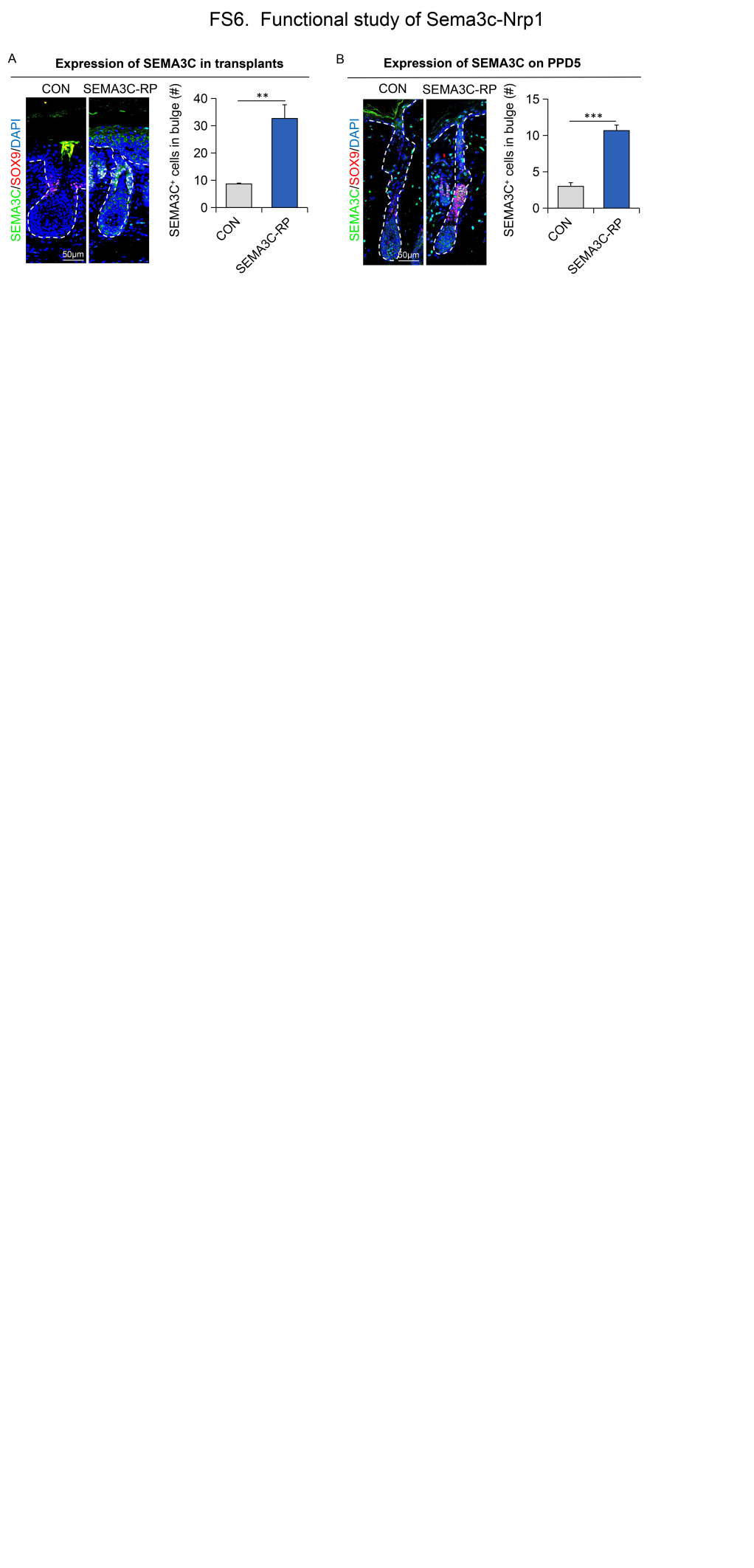
**

**Figure S6. Functional study of the SEMA3C-NRP1 axis**
A. Immunofluorescence staining for SEMA3C, SOX9, and DAPI, with quantification of SEMA3C/SOX9 co-expressing cells in skin organoids from the control and SEMA3C-activated groups at 9 days post-transplantation. N ≥ 3; **p < 0.01, scale bar = 50 μm.
B. Immunofluorescence staining for SEMA3C, SOX9, and DAPI, with quantification of SEMA3C/SOX9 co-expressing cells in skin organoids from the control and SEMA3C-activated groups on Day 5 in plucked mice. N ≥ 3; ***p < 0.001, scale bar = 50 μm.

**
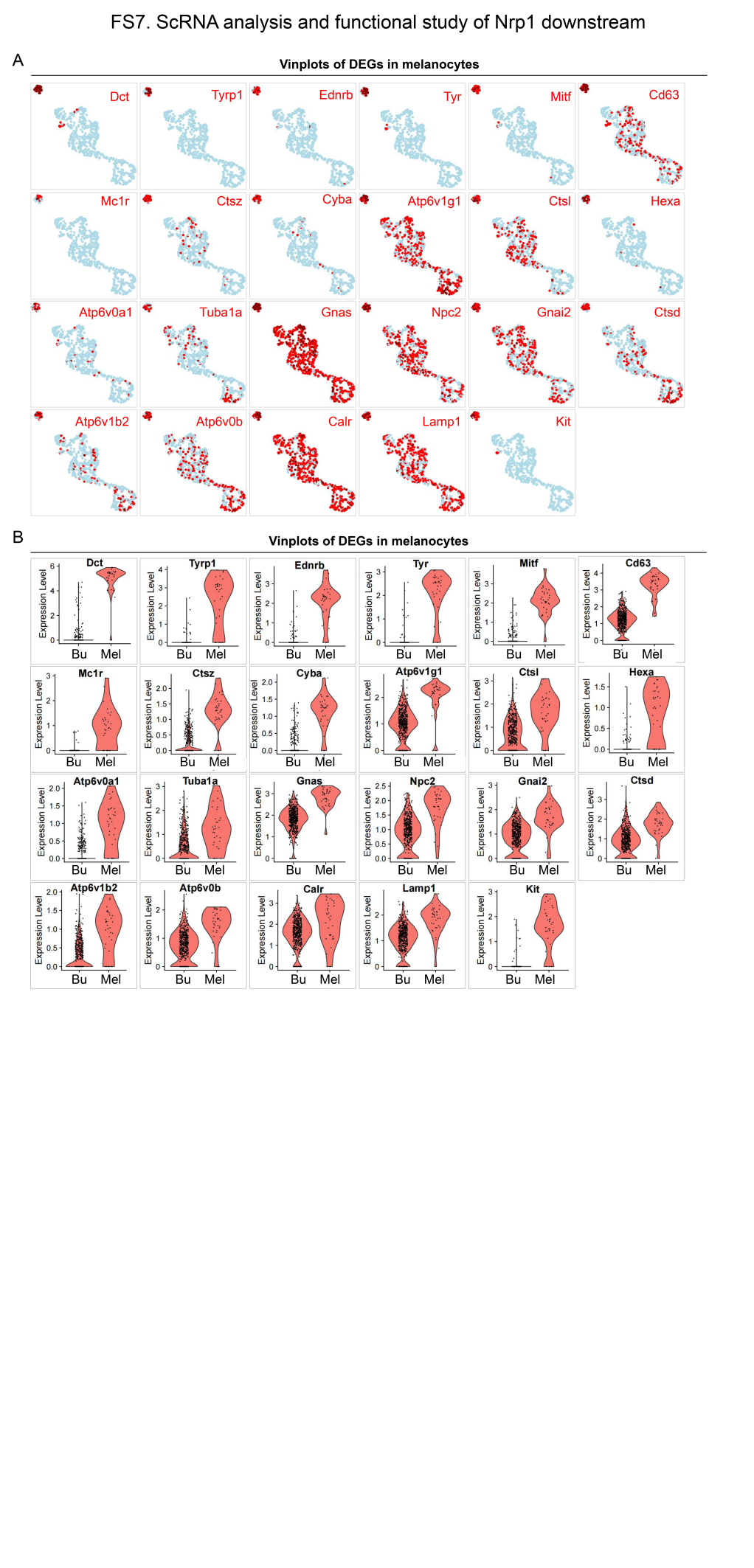
**

**Figure S7. scRNA analysis and functional study of NRP1 downstream pathways**
A. Feature plots of differentially expressed genes for the top three enriched pathways.
B. Violin plots showing top three pathway-specific differentially expressed genes.
